# Multifunctional biosynthesized magnetosome for multimodal imaging and combined therapy of tumor

**DOI:** 10.1016/j.mtbio.2024.101429

**Published:** 2024-12-24

**Authors:** Xiaoqing Han, Xingbo Wang, Jiao Yan, Panpan Song, Yanjing Wang, Yaqing Kang, Abdur Rauf, Haiyuan Zhang

**Affiliations:** a Key Laboratory of Molecular Epigenetics of the Ministry of Education (MOE), Northeast Normal University, Changchun, 130024, China; b Laboratory of Chemical Biology, Changchun Institute of Applied Chemistry, Chinese Academy of Sciences, Changchun, 130022, China; c School of Biomedical Engineering & The First Affiliated Hospital, Guangzhou Medical University, Guangzhou, 511436, China; d Department of Chemistry, University of Swabi, Ambar, 23430, Pakistan

**Keywords:** Magnetosome, Tumor-associated macrophages, Photoimmunotherapy, Repolarization

## Abstract

The large recruitment of tumor-associated macrophages and low exposure of tumor-associated antigens in tumor microenvironment have severely suppress the efficacy of anti-tumor immunotherapy. Herein, biosynthesized magnetosome (Mag) from bacteria was loaded with photothermal/photodynamic agent/near infrared (NIR) fluorescence dye (IR780) and further modified with lipid-PEG-c(RGDyK) through biomembrane, forming _I_Mag^RGD^ for fluorescence imaging, magnetic resonance imaging, immunotherapy and photodynamic/photothermal therapy. After intravenous injection into B16F10 tumor-bearing mice, _I_Mag^RGD^ could efficiently accumulate in tumor tissues based on near infrared (NIR) fluorescence and magnetic resonance dual-modality imaging, and repolarize tumor-associated macrophages (TAMs) from M2 phenotype to M1 phenotype, significantly improving the effect of tumor immunotherapy. Moreover, photothermal and photodynamic effect of IR780 could kill tumor cells and elicit immunogenic cell death to mediate anti-tumor immunity, promoting dendritic cells (DCs) maturation and then activating specific effector T cells to further eliminate tumor cells. This study provides a new approach for reversing the activity of tumor immunosuppressive microenvironment and strengthening the efficiency of tumor photoimmunotherapy.

## Graphical abstract

**Figure:**
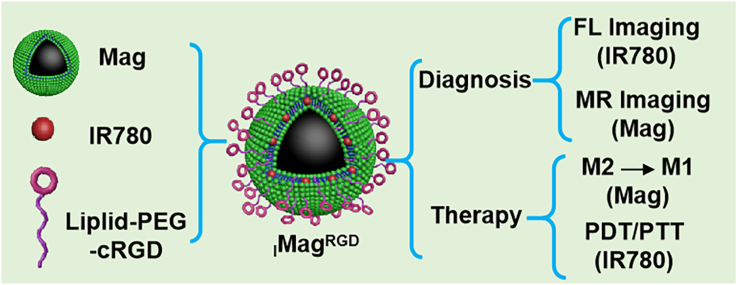

## 1 Introduction

Clinical success of immune checkpoint blockade and chimeric antigen receptor T cell therapy indicates that cancer immunotherapy has made great progress [1]. However, the efficacy of immunotherapy for most tumors is limited due to the immunosuppressive tumor microenvironment [2] that can destroy the activation of immune cells and even convert them into immunosuppressive cells [[3], [4], [5]]. Tumor-associated macrophages (TAMs) represent the most abundant population type of tumor-infiltrating immune cells that construct immunosuppressive tumor microenvironment [6]. Most of TAMs exhibit M2 phenotype, which promote tumor progression and metastasis through stimulating angiogenesis, increasing tumor cell migration, invasion and intravasation as well as suppressing antitumor immunity [7,8]. Therefore, various therapeutic strategies have been developed to transform TAMs from the protumor M2 phenotype to the antitumor M1 phenotype. Small molecules and monoclonal antibodies (mAbs), such as bisphosphonates [9], BLZ945 [10], anti-colony-stimulating factor-1 receptor (CSF1R) mAbs [11] and anti-cluster of differentiation 47 (CD47) mAbs [12], have been used to activate the repolarization of M2 phenotype TAMs. To further improve drug accumulation at the tumor site, various nanomaterials, such as lipid-based nanomaterials, polymeric nanoparticles, manganese dioxide nanoparticles and iron-based nanomaterials, have been designed as carriers [13]. Iron oxide nanomaterials have recently been reported the capability of promoting the repolarization of TAMs from M2 phenotype to M1 phenotype, which is not affected by their surface modification [14,15]. Iron oxide nanomaterials provide a guarantee for the combination of immunotherapy and other tumor therapeutic strategies. However, chemosynthetic iron oxide nanomaterials have acute and chronic toxicities even after surface biocompatible modification, unsuitable for in vivo immunotherapy [16,17]. For example, encapsulation of iron oxide nanoparticles with polyethylene glycol (PEG) chelated D-mannose increases cellular uptake, but it causes PEG-related allergic reactions [18,19]. Recently, it has been reported that the magnetosome (Mag) biomineralized from magnetotactic bacteria contains iron oxide nanoparticle with biomembrane, which shows high biocompatibility and easy surface modification with antibodies, photosensitizers and drugs through biomembrane [[20], [21], [22], [23]]. These unique features potentially enable bacterial Mag to be used in repolarization of M2 TAMs towards M1 phenotype.

After this repolarization of TAMs in the tumor microenvironment, M1 macrophages would directly kill tumor cells by secreting interferon gamma (IFN-γ) and tumor necrosis factor alpha (TNF-α) *etc* [24]. On the basis, further increase of effective antigen exposure in solid tumors will promote the infiltration of cytotoxic T lymphocytes and effector T cells to enhance the efficacy of tumor immunotherapy [5]. Among various strategies for increasing tumor antigen exposure, photodynamic therapy (PDT) and photothermal therapy (PTT) with spatiotemporal control and minimal invasions have aroused considerable interest. PDT converts oxygen into highly active singlet oxygen (^1^O_2_) by illuminating a photosensitizer, and PTT produces heat energy by converting light energy, both of which can damage tumor cells, induce immunogenic cell death (ICD) and release tumor cell debris. The tumor cell debris acts as tumor-associate antigens (TAAs) to promote dendritic cells (DCs) maturation, followed by education and priming of cytotoxic T cells, causing anti-tumor immune responses [25,26]. However, the anti-tumor immune effect induced by phototherapy is not enough to reverse the tumor microenvironment from immunosuppressive to immunostimulative [27,28]. Therefore, the combination of TAM repolarization and phototherapy potentially will overcome this deficiency, becoming a desirable strategy for efficient antitumor therapy.

**Fig. 1:**
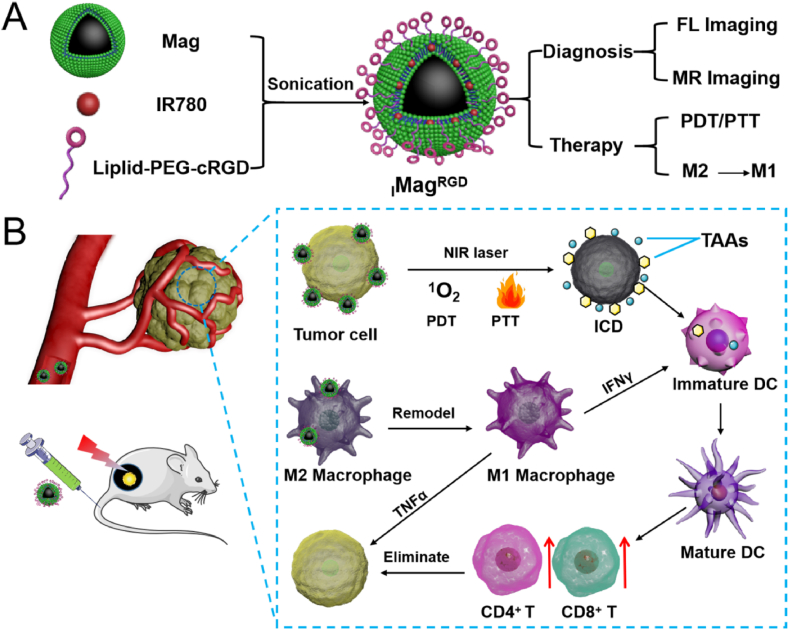
**Schematic illustration of**_**I**_**Mag**^**RGD**^**preparation and functions for photoimmunotherapy**. (A) Preparation of _I_Mag^RGD^ through surface modification with IR780 and c(RGD). (B) _I_Mag^RGD^ promotes TAMs repolarization from M2 to M1 phenotype and implementes PTT/PDT to induce ICD of tumor cells and release TAAs, resulting in the maturation of DCs and infiltration of effect T lymphocytes.

IR-780, which is a lipophilic near infrared (NIR) fluorescent probe and can also generate ^1^O_2_ and heat under 808 nm laser irradiation [29], has been used for NIR fluorescence imaging and PDT/PTT therapy, however, its poor water solubility and low tumor-targeting ability dramatically limit its clinical application. Biomembrane of Mag is composed of a phospholipid bilayer structure [30], which can be used for embedment of lipophilic molecule and lipid chain through hydrophobic-hydrophobic interaction [31,32]. In the present study, lipophilic IR-780 was embedded in the lipophilic phospholipid bilayer of Mag, forming _I_Mag, and a tumor-targeting peptide (cyclo (Arg-Gly-Asp-D-Tyr-Lys) [33] which is a c(RGDyK)) with a lipid-PEG tail, was further modified on the surface of _I_Mag through inserting its lipid tail into lipophilic phospholipid bilayer, forming _I_Mag^RGD^ (Fig. 1A). Once intravenously injected into B16F10 tumor-bearing mouse model, _I_Mag^RGD^ could efficiently accumulate tumor tissues based on cRGD property and repolarize TAMs from M2 phenotype to M1 phenotype through iron oxide core, exhibiting tumor immunotherapeutic effect. Under NIR laser irradiation, _I_Mag^RGD^ could kill tumor cells and elicit ICD to initiate an effective immune response against endogenous tumor antigens (Fig. 1B). Besides, NIR fluorescence and magnetic properties of _I_Mag^RGD^ could be used to track its biodistribution through fluorescence and magnetic resonance dual-modality imaging. Overall, _I_Mag^RGD^ holds great potential in tumor photoimmunotherapy.

## 2 Results and discussion

1. Synthesis and Characterization of _I_Mag^RGD^

**Fig. 2:**
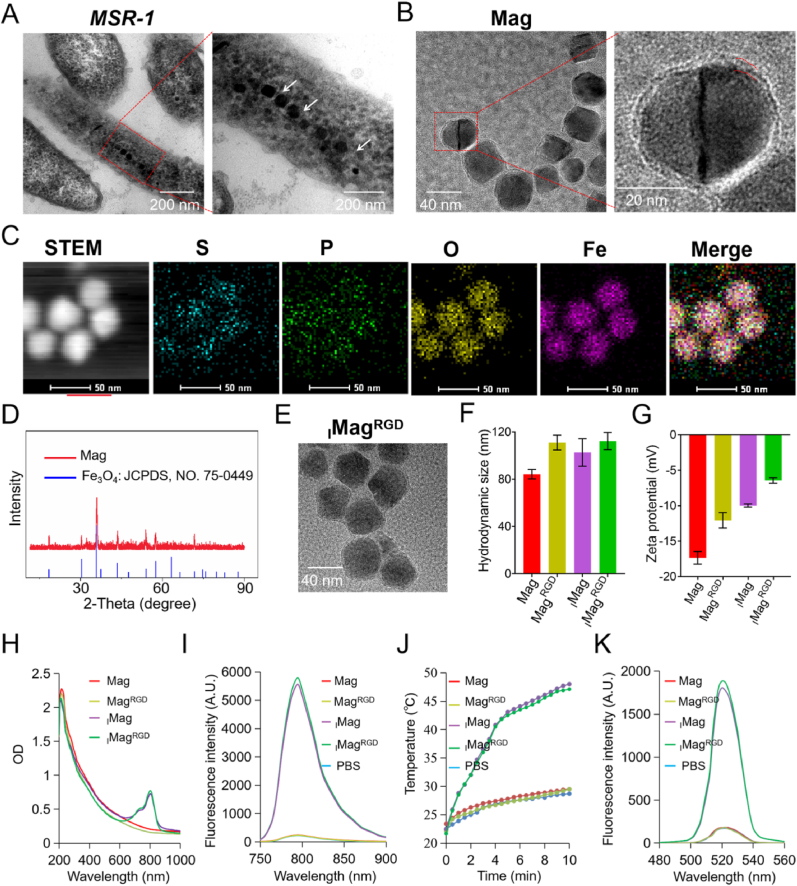
**Characterization of**_**I**_**Mag**^**RGD**^. (A) TEM image of *MSR-1*, where the arrows indicate the location of Mag. (B) TEM image of Mag, where the biomembrane could be observed on the nanoparticle. (C) EDS elemental mapping images of Mag. (D) XRD pattern of Mag. (E) TEM image of _I_Mag^RGD^. (F–I) Hydrodynamic sizes (F), Zeta potential (G), UV–Vis spectroscopy (H) and fluorescence emission spectra excited at 740 nm (I) of Mag, Mag^RGD^, _I_Mag and _I_Mag^RGD^. (J, K) Temperature elevation curves (J) and SOSG fluorescence emission spectra (K) of Mag, Mag^RGD^, _I_Mag and _I_Mag^RGD^ in PBS under 808 nm laser irradiation (0.75 W cm^−2^, 5 min). SOSG fluorescence assay was used to assess the ^1^O_2_ generation performance. Data are expressed as means ± SD (n = 3).

*Magnetospirillum gryphiswaldense MSR-1* was cultured in schleifer liquid medium containing ferric citrate for producing bacterial Mag [34]. After 48 h of culture, transmission electron microscopy (TEM) images of *MSR-1* cells revealed the formation of Mag in *MSR-1* (Fig. 2A) which was arranged in chain-like morphology. After collection and purification of Mag, TEM image revealed the membrane surrounding the nanoparticles, indicating that the collection and purification procedure does not damage the biomembrane of Mag (Fig. 2B). Energy dispersive X-ray (EDS) elemental mapping images (Fig. 2C) of Mag clearly indicated that Mag contained sulfur (S, blue), phosphorus (P, green), and oxygen (O, yellow) elements, which are the major elements of the proteins and phospholipids of the membrane. X-ray diffraction (XRD) matched well with the crystalline structure of Fe_3_O_4_ (JCPDS 75–0449) that confirmed the component of Fe_3_O_4_ in Mag (Fig. 2D). Then, Mag was loaded with both IR780 and lipid-PEG-c(RGDyK) through sonication to form _I_Mag^RGD^. TEM image of _I_Mag^RGD^ showed the morphology and size did not change before and after the modifications (Fig. 2E). Similarly, Mag loaded with lipid-PEG-c(RGDyK) (Mag^RGD^) and Mag loaded with IR780 (_I_Mag) were prepared for comparison. The morphology and sizes of Mag^RGD^ and _I_Mag were examined through TEM, and no significant difference was observed between them (Fig. S1). Dynamic light scattering (DLS) measurements showed the hydrodynamic sizes of _I_Mag^RGD^, Mag^RGD^ and _I_Mag in phosphate buffered saline (PBS) were 112.3 ± 5.9, 111 ± 5.1 nm and 102.7 ± 3.3, respectively, which was slightly larger than that of Mag (84.3 ± 3.3 nm) (Fig. 2F). The loading of IR780 or lipid-PEG-c(RGDyK) reduced the zeta potential of Mag, as shown in _I_Mag^RGD^, Mag^RGD^ and _I_Mag (Fig. 2G). The characteristic absorption peak of IR780 at 810 nm could be observed in ultraviolet–visible (UV–Vis) spectroscopy of _I_Mag and _I_Mag^RGD^ (Fig. 2H) and the fluorescence emission peak of IR780 was observed at 800 nm in their fluorescence spectra (Fig. 2I), suggesting that hydrophobic IR780 has been successfully embedded into the biomembrane of Mag. The loading capacities of IR780 in _I_Mag^RGD^ and _I_Mag were determined as 10.8 % and 10.6 %, respectively, based on the absorption property of IR780. Next, c(RGDyK) contents in _I_Mag^RGD^ and Mag^RGD^ were determined as 8.3 % and 8.2 %, respectively, based on arginine measurement [35]. Moreover, Fe contents in _I_Mag^RGD^, _I_Mag, Mag^RGD^ and Mag were determined as 5.7, 6.5, 6.3 and 7.1 %, respectively, based on inductively coupled plasma-optical emission spectroscopy analysis. Based on the previously reported calculation method [36], the lipid inserting efficiencies of _I_Mag^RGD^ and Mag^RGD^ were determined as 335 and 295 lipid-PEG-cRGD per each particle, respectively.

Photothermal performance of various types of Mags was detected by their temperature elevation profiles in PBS. Both _I_Mag and _I_Mag^RGD^ increased the temperature of 26 °C under 808 nm laser irradiation at 0.75 W cm^−2^ for 5 min. However, under the same conditions, Mag and Mag^RGD^ only increased the temperature of 6 °C (Fig. 2J), close to the profile of PBS. Then, the photodynamic performance of these Mag was evaluated by singlet oxygen sensor green (SOSG) fluorescence measurement. There was much reactive oxygen species (ROS) generation observed on _I_Mag and _I_Mag^RGD^ under 808 nm laser irradiation (0.75 W cm^−2^, 5 min), but only a little ROS generation was observed in Mag and Mag^RGD^ (Fig. 2K). Without 808 nm laser irradiation, all these Mags showed weak fluorescence intensities, similar to that of PBS (Fig. S2). The above results demonstrate that _I_Mag^RGD^ has good photothermal and photodynamic performance, beneficial for simultaneous PTT/PDT treatment.

2. Photothermal/photodynamic cellular effect of _I_Mag^RGD^ on tumor cell injury and DC maturation

**Fig. 3:**
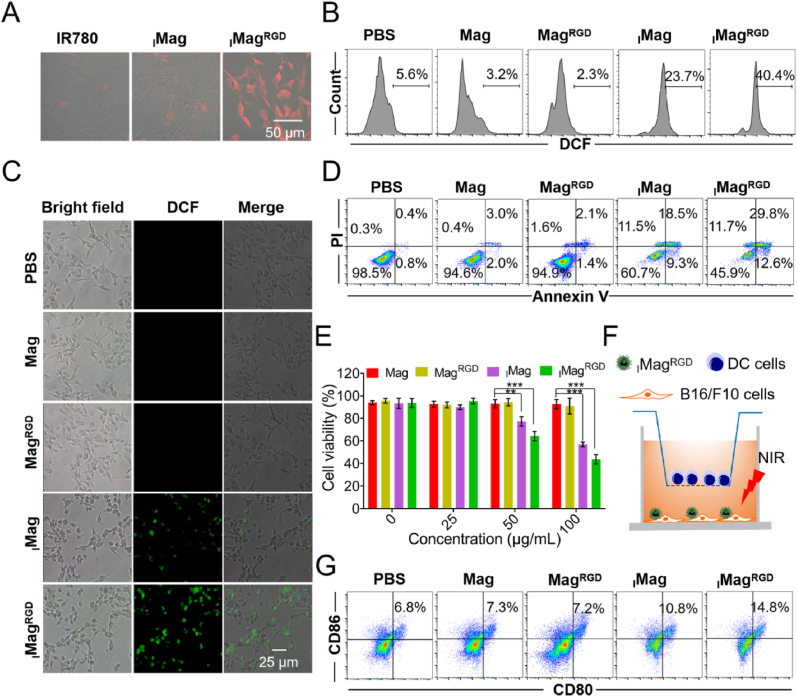
**Photothermal/photodynamic effects of**_**I**_**Mag**^**RGD**^**on B16/F10** **cell injury and DC activation.** (A) IR780-based NIR fluorescence microscopy images of B16/F10 cells to show the uptake of free IR780, _I_Mag and _I_Mag^RGD^ after 3 h treatment; (B and C) DCF-based flow cytometry analysis (B) and fluorescence microscopy images (C) for the intracellular ROS level of B16/F10 cells. (D) Flow cytometry analysis for apoptotic B16/F10 cells based on Annexin-FITC/PI staining. (E) MTT-based viability assessment of B16/F10 cells after 24 h of treatment with different concentrations of different Mags (equivalent to Fe_3_O_4_ content) under 808 nm laser irradiation. (F) Establishment of DCs/B16F10 transwell system. (G) Flow cytometry analysis for the percentage of mature DCs (CD86^+^CD80^+^) as illustrated in F. For B to D and G, B16/F10 cells were treated with of different types of Mags (equivalent to 100 μg mL^−1^ Fe_3_O_4_ content) for 6 h, followed by an 808 nm laser irradiation (0.75 W cm^−2^, 5 min). Data are expressed as means ± SD (n = 3).

The cells uptake of _I_Mag^RGD^ was investigated in B16/F10 cells by fluorescence microscopy. After 3 h of incubation, there was only a slight IR780 fluorescence observed in cells treated with free IR780 or _I_Mag. In contrast, _I_Mag^RGD^-treated cells presented significantly stronger fluorescence (Fig. 3A), suggesting the specific uptake of _I_Mag^RGD^ by tumor cells, which is because that c(RGDyK) can bind with α_V_β_3_ integrin receptors on the surface of tumor cell membrane. The intracellular ROS level of B16/F10 cells treated with various types of Mags with or without NIR laser irradiation was evaluated by flow cytometry and fluorescence microscopy based on 2′,7′-Dichlorodihydrofluorescein diacetate (DCFH-DA) probe. With NIR laser irradiation, cells treated with _I_Mag and _I_Mag^RGD^ showed stronger green fluorescence than those treated with PBS, Mag and Mag^RGD^ (Fig. 3B–S3 and 3C), indicating that the IR780 loading can greatly elevate intracellular ROS level. Moreover, the ROS level in _I_Mag^RGD^-treated cells was higher than that of _I_Mag-treated cells, which is due to the targeting ability of _I_Mag^RGD^ to B16/F10 cells (Fig. 3B–S3 and 3C). Without NIR laser irradiation, all the cells in various groups showed little green fluorescence (Figs. S4A–C). These results suggest that NIR laser can promote photothermal/photodynamic effects to enhance the ROS generation. Then, the apoptosis of B16/F10 cells treated with various types of Mags with or without NIR laser irradiation was evaluated by flow cytometry analysis. The results showed that the accumulative percentage of early apoptotic, late apoptotic and necrotic B16/F10 cells treated with _I_Mag^RGD^ was the highest among various groups under NIR laser irradiation (Fig. 3D and S5). But without NIR laser irradiation, all the cells in various groups could hardly induce apoptosis and necrosis (Figs. S6A and B). The therapeutic effects of various types of Mags in B16F10 cells were further evaluated by detection of cell viability through 3-(4,5-dimethylthiazolyl-2)-2,5-diphenyltetra-zolium bromide (MTT) assay. With NIR laser irradiation, there was no noticeable change in the viability of cells treated with PBS, Mag or Mag^RGD^, however, treatment with _I_Mag or _I_Mag^RGD^ gradually decreased the cell viability with the rise of Mag concentration, where _I_Mag^RGD^ showed the higher cytotoxicity than _I_Mag (Fig. 3E). Without NIR laser irradiation, all these types of Mags had no effect on the cell viability (Fig. S7). Taken together, all above results demonstrate that _I_Mag^RGD^ has the significant inhibition ability on tumor cell growth based on its PTT and PDT performance.

Recent studies found that PTT/PDT could induce ICD to improve anti-tumor immunogenicity [37]. To investigate the ability of _I_Mag^RGD^ to promote DCs maturation, a DCs/B16F10 transwell system was established, where DCs were cultured in the upper chamber and B16F10 cells in the lower chamber (Fig. 3F). B16F10 cells were incubated with Mag, Mag^RGD^, _I_Mag or _I_Mag^RGD^ for 6 h in the lower chamber, followed by an 808 nm laser irradiation (0.75 W cm^−2^, 5 min). After another 18 h incubation, DCs in the upper chamber was collected for detection of DCs maturation by flow cytometry. The result showed that _I_Mag and _I_Mag^RGD^ could induce more amounts of mature DCs (CD86^+^CD80^+^) than Mag and Mag^RGD^, where _I_Mag^RGD^ showed more excellent performance than _I_Mag due to the targeting ability of c(RGDyK) (Fig. 3G and S8). Maturation of DCs is accompanied by the upregulation of the costimulatory molecules (e.g., CD80 and CD86) as well as secretion of proinflammatory cytokines [38,39]. Then, the proinflammatory cytokines including interleukin 12 (IL-12) and TNF-α [40,41] as well as anti-inflammatory cytokines including interleukin 6 (IL-6) and interleukin 10 (IL-10) [42] in the supernatant of the DCs/B16F10 transwell system were detected. Under 808 nm laser irradiation (0.75 W cm^−2^, 5 min), compared with PBS group, treatment with Mag and Mag^RGD^ showed little influence on TNF-α, IL-12, IL-10 and IL-6 levels, however, treatment with _I_Mag and _I_Mag^RGD^ could significantly induce increased TNF-α and IL12 levels as well as decreased IL-10 and IL-6 levels. _I_Mag^RGD^ group secreted the highest levels of TNF-α and IL12 as well as the lowest levels of IL-10 and IL-6 among all the groups due to the targeting ability of c(RGDyK) and the loading of IR780 (Figs. S9A–D). Furthermore, without NIR laser irradiation, there was no noticeable changes in the amounts of mature DCs (CD86^+^CD80^+^) and the levels of IL-12, TNF-α, IL-6 and IL-10 in different treated groups compared with PBS group (Figs. S10A–F), suggesting _I_Mag^RGD^-mediated PTT/PDT can elicit ICD and promote DCs maturation.

3. Macrophage repolarization by _I_Mag^RGD^

**Fig. 4:**
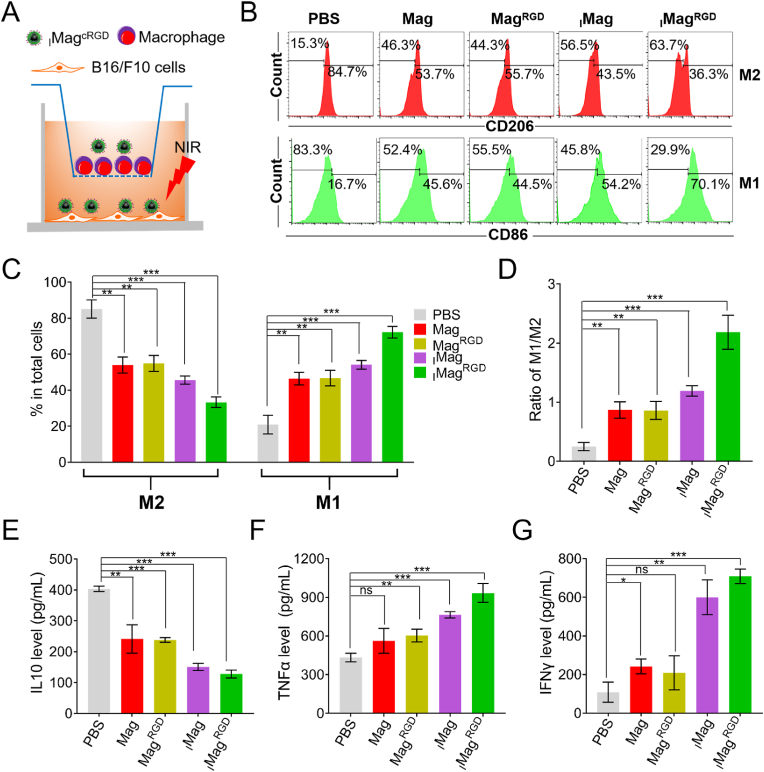
**Macrophages repolarization by**_**I**_**Mag**^**RGD**^**in RAW264**.**7** **cells.** (A) Establishment of RAW264.7/B16F10 transwell system. (B) Flow cytometric analysis of CD86 and CD206 expression of RAW264.7 cells treated with Mag, Mag^RGD^, _I_Mag or _I_Mag^RGD^ (equivalent to 100 μg mL^−1^ Fe_3_O_4_) in RAW264.7/B16F10 transwell system with 808 nm laser irradiation (0.75 W cm^−2^, 5 min); RAW264.7 cells were first induced into M2 macrophages by IL-4 and then plated in transwell system. (C) Quantitative analysis of the percentages of M1 and M2 in Fig. 4B. (D) M1/M2 ratio in Fig. 4B. (E–G) ELISA analysis of the levels of IL-10 (E), TNF-α (F) and IFN-γ (G) in the supernatant of the RAW264.7/B16F10 transwell system in Fig. 4A. Data are expressed as means ± SD (n = 3).

It has been known that iron oxide nanoparticles can activate macrophages and repolarize M2 TAMs toward M1 TAMs [43]. ROS generation in tumor microenvironment not only induces cancer cell apoptosis but also polarizes TAMs, improving the cancer therapy [44]. Therefore, to investigate whether _I_Mag^RGD^ had an immunological effect on TAM polarization, a RAW264.7/B16F10 transwell system was established, where RAW264.7 cells that were induced by interleukin 4 (IL-4) into M2 macrophages were cultured in the upper chamber and B16F10 cells in the lower chamber (Fig. 4A). Mag, Mag^RGD^, _I_Mag or _I_Mag^RGD^ was added to both chambers for 6 h, followed by an 808 nm laser irradiation (0.75 W cm^−2^, 5 min) on the lower chamber. After another 18 h of incubation, the treated RAW264.7 cells were collected and labeled with CD86/PE and CD206/APC antibodies. Flow cytometry analysis revealed all the Mag, Mag^RGD^, _I_Mag and _I_Mag^RGD^ compared with PBS remarkably up-regulated the expression of CD86 and down-regulated the expression of CD206 (Fig. 4B and C), where _I_Mag^RGD^ group was the most effective and induced the highest ratio of M1/M2 among all the groups (Fig. 4D). Besides, the supernatant of transwell system was collected to detect the levels of TNF-α and IFN-γ (M1 marker) and IL-10 (M2 marker). Treatment with Mag, Mag^RGD^, _I_Mag or _I_Mag^RGD^ apparently decreased the level of IL-10 (Fig. 4E), but obviously increased the secretion levels of TNF-α and IFN-γ (Fig. 4F and G). Also, _I_Mag^RGD^ group secreted the lowest level of IL-10 and highest level of TNF-α and IFN-γ among all the groups. In comparison, without NIR laser irradiation, all these Mag groups compared with PBS group could slightly change the expressions of CD86 and CD206 (Figs. S11A and B), and the levels of IL-10, TNF-α and IFN-γ (Figs. S12A–C). These results indicate that various types of Mags can induce the repolarization of M2 macrophages to M1 phenotype, which can be further effectively potentiated by _I_Mag^RGD^ under NIR laser irradiation due to IR780 and c(RGDyK) modification.

4. Tumor imaging ability of _I_Mag^RGD^ in vivo

**Fig. 5:**
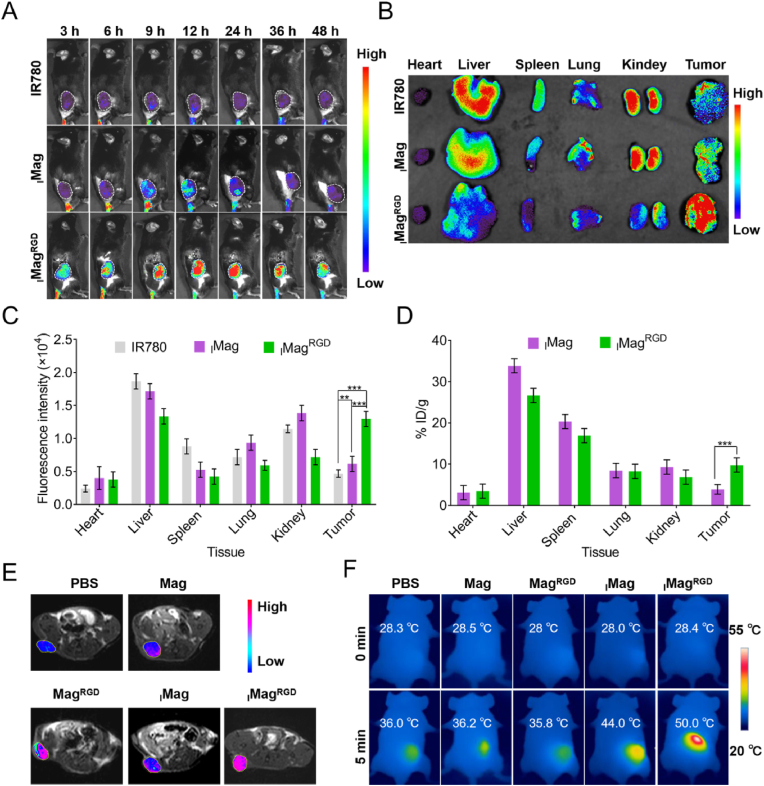
**Tumor imaging ability of**_**I**_**Mag**^**RGD**^**in B16/F10 tumor-bearing mice.** (A) Fluorescence imaging of mice after intravenous injection with free IR780, _I_Mag or _I_Mag^RGD^ (equivalent to 0.25 mg kg^−1^ IR780) at different time points. The tumor sites were circled with white lines. (B) Ex vivo fluorescence imaging of tumor and major tissues of B16/F10 tumor-bearing mice at 48 h post-injection. (C) Quantification of fluorescence intensity of tumor and major tissues in (B). (D) ICP-OES analysis for biodistribution of _I_Mag and _I_Mag^RGD^ in mice at 48 h postinjection based on Fe element. (E) T1-weighted MR images of tumors after 12 h post-injection of PBS, Mag, Mag^RGD^, _I_Mag or _I_Mag^RGD^ (equivalent to 10 mg kg^−1^ Fe_3_O_4_). (F) Infrared thermal images of tumor region that was exposed to 808 nm laser for 5 min after 12 h post-injection of PBS, Mag, Mag^RGD^, _I_Mag or _I_Mag^RGD^ (equivalent to 10 mg kg^−1^ Fe_3_O_4_). Data are expressed as means ± SD (n = 5).

To investigate the performance of _I_Mag^RGD^ for NIR fluorescence imaging in vivo, B16/F10 tumor-bearing C57BL/6J mice were intravenously injected with free IR780, _I_Mag or _I_Mag^RGD^ (equivalent to 0.25 mg kg^−1^ IR780). The biodistribution of _I_Mag or _I_Mag^RGD^ at 3–48 h post-injection was examined by whole-body fluorescence imaging system based on IR780 fluorescence. The fluorescence signal of IR780 in tumor regions reached the strongest at 12 h post-injection in IR780, _I_Mag or _I_Mag^RGD^ group, and gradually decayed over time. Noticeably, _I_Mag^RGD^ group displayed the strongest fluorescence intensity and had the longest duration (at least 48 h) in tumor tissue among all the groups (Fig. 5A). At 48 h post-injection, all mice were sacrificed, and tumors and major organs were harvested for acquisition of ex vivo fluorescence images and quantification (Fig. 5B and C). It was found that IR780 and _I_Mag groups showed higher IR780 fluorescence intensities in liver and kidney than other tissues, while _I_Mag^RGD^ group had higher intensity in liver and tumor. The additional tumor tissue sections at 48 h post-injection of IR780, _I_Mag or _I_Mag^RGD^ were prepared for immunofluorescence staining on macrophages (F4/80 labeled by FITC) and tumor cells (HMB45 (marker of B16F10 cells) labeled by FITC), respectively. The results showed that _I_Mag^RGD^ colocalized with both macrophages (Fig. S13A) and tumor cells (Fig. S13B). Moreover, the corresponding inductively coupled plasma optical emission spectroscopy (ICP-OES) analysis based on Fe element also proved that _I_Mag^RGD^ compared with _I_Mag could more significantly accumulate in tumor (Fig. 5D), which should be attributed to the tumor-targeting capability of c(RGDyK). All above results confirm that _I_Mag^RGD^ can be used as a promising fluorescence imaging probe for tumor diagnosis. Furthermore, the performance of _I_Mag^RGD^ for in vivo magnetic resonance (MR) imaging was investigated. The MR images were taken at 12 h post-injection with PBS, Mag, Mag^RGD^, _I_Mag or _I_Mag^RGD^ using a Bruker 7.0 T MR imaging system. There were sharper signal intensities in the tumor regions of Mag^RGD^ and _I_Mag^RGD^ groups compared with PBS, Mag and _I_Mag groups (Fig. 5E). These results were consistent with above fluorescence imaging results, demonstrating the promising MR imaging performance of Mag^RGD^. In addition, the real-time temperature variation of tumor tissue was evaluated by infrared thermal imaging system at 12 h post-injection. Upon an 808 nm laser irradiation, the temperature in tumor tissue of _I_Mag^RGD^ group rapidly rose from 28.4 to 50.0 °C within 5 min, while the temperature in tumor tissue of _I_Mag group increased from 28.0 °C to 44.0 °C, and the temperature in tumor tissue of PBS, Mag and Mag^RGD^ groups only exhibited a slight increase (Fig. 5F). It further consolidates that _I_Mag^RGD^ can more efficiently accumulate in tumor tissue for photothermal/photodynamic therapy.

5. In vivo antitumor efficacy of _I_Mag^RGD^

**Fig. 6:**
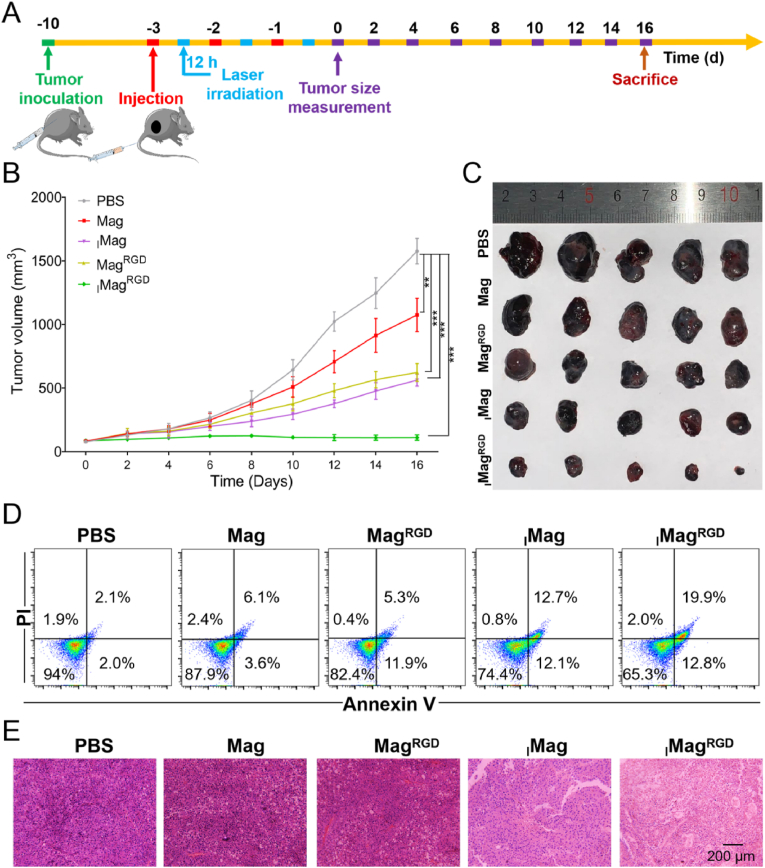
**In vivo therapeutic efficiency of**_**I**_**Mag**^**RGD**^**with NIR laser irradiation.** (A) Schematic illustration of tumor model establishment and therapy process. (B) Tumor volume growth of mice during 16 days of treatment. (C) Photograph of tumor tissues at the end of 16 days of treatment. (D) Flow cytometry analysis for apoptotic cells in tumor at the end of 16 days of treatment based on Annexin-FITC/PI staining. (E) H&E staining of tumor tissue at the end of 16 days of treatment. For (B) to (E), B16/F10 tumor-bearing mice were intravenously injected with PBS, Mag, Mag^RGD^, _I_Mag or _I_Mag^RGD^ (equivalent to 10 mg kg^−1^ Fe_3_O_4_) with NIR laser irradiation (808 nm laser, 0.75 W cm^−2^, 5 min). Data are expressed as means ± SD (n = 5).

The antitumor efficacy of _I_Mag^RGD^ was investigated using B16/F10 tumor-bearing mice in vivo. As shown in Fig. 6, Fig. 1 × 10^6^ B16/F10 cells were subcutaneously injected into the mice. When the tumor volume reached about 100 mm^3^, the mice were randomly divided into 5 groups. Then, mice were intravenously injected with PBS, Mag, Mag^RGD^, _I_Mag or _I_Mag^RGD^ (equivalent to 10 mg kg^−1^ Fe_3_O_4_) on -3d, -2d and -1d, and exposed to 808 nm laser (0.75 W cm^−2^, 5 min) at 12 h after each injection. The tumor volume was measured every other day for 16 days. Fig. 6B shows the change of tumor volume over time. Compared with the treatment with PBS, the treatment with Mag, Mag^RGD^, _I_Mag or _I_Mag^RGD^ could suppress tumor growth to a certain extent under NIR laser irradiation, where the treatment with _I_Mag^RGD^ showed the strongest tumor growth inhibition efficiency. Fig. 6C shows the visual images of tumor tissues at the end of 16 days of treatments. However, without NIR laser irradiation, treatment with Mag, Mag^RGD^, _I_Mag or _I_Mag^RGD^ only slightly inhibited the tumor growth (Figs. S14A and B), which might be attributed to macrophage repolarization of Mags. Then, the percentages of apoptotic cells in tumor tissues of different groups were examined by flow cytometry. With NIR laser irradiation, Fig. 6D and S15 show that the total percentage of early and late apoptotic cells in the tumor tissues of _I_Mag^RGD^ group was the highest among all the groups. But without 808 nm laser irradiation, all these Mags could induce very low percentages of early and late apoptosis (Figs. S16A and B). Moreover, H&E staining results of tumor tissues confirmed the most severe damage on tumor tissue (including karyotheca lysis, severe necrosis and nucleolus vanishing) in _I_Mag^RGD^ group with NIR laser irradiation (Fig. 6E). Little damage was observed in tumor tissues of all the groups without NIR laser irradiation (Fig. S17). Therefore, above results suggest that _I_Mag^RGD^ can serve as an ideal therapeutic agent for tumor photoimmunotherapy in the clinical trials in the future.

In vivo biocompatibility of _I_Mag^RGD^ was further investigated. During the treatments, the body weights of all treated groups showed no significant loss and displayed a slight upward trend (Fig. S18). Besides, there was no obvious abnormality in the H&E staining images of major organs (Fig. S19) and serum biochemical indices (including aspartate aminotransferase (AST), alanine aminotransferase (ALT), creatinine (CREA) and blood urea nitrogen (BUN)) of mice (Figs. S20A–D) in all groups, demonstrating the excellent biocompatibility of _I_Mag^RGD^ for tumor therapy.

6. In vivo M2-to-M1 macrophage repolarization and antitumor immunity activation capability of _I_Mag^RGD^

**Fig. 7:**
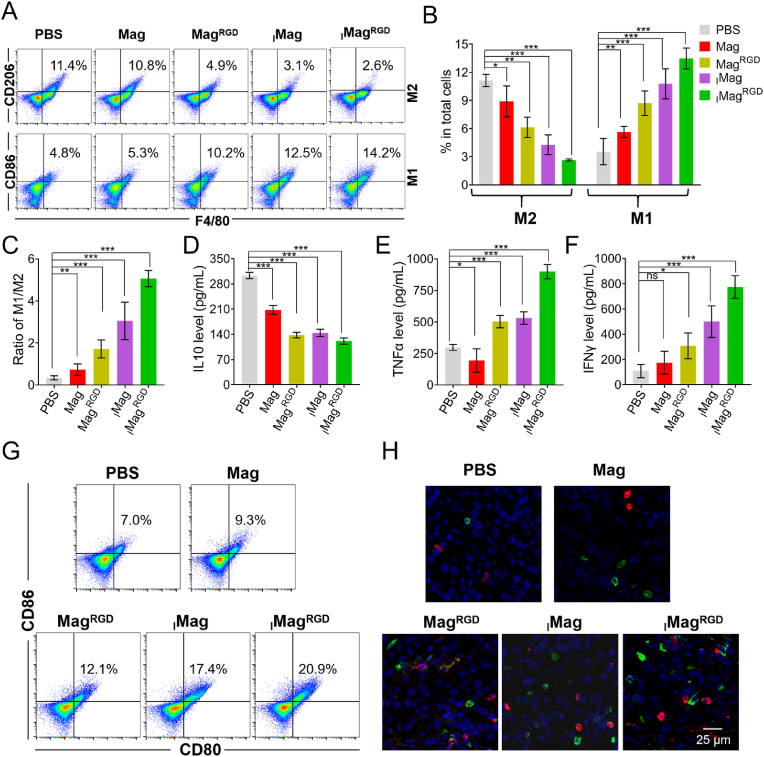
**Relieved immune-suppressive microenvironment by**_**I**_**Mag**^**RGD**^**with NIR laser irradiation**. (A) Flow cytometric analysis of the percentages of M2-type macrophages (F4/80^+^CD206^+^) and M1-type macrophages (F4/80^+^CD86^+^) in tumor tissues. (B) Quantitative analysis of Fig. 7B. (C) Ratio of M1/M2 in Fig. 7B. (D–F) ELISA analysis for the levels of IL-10 (D), TNF-α (E) and IFN-γ (F) in tumor tissues. (G) Flow cytometry analysis for the percentage of mature DCs in draining lymph node. (H) Immunofluorescence images of tumors stained by T helper cells (red, CD4) and cytotoxic T lymphocytes (green, CD8) markers. For (A) to (H), B16/F10 tumor-bearing mice were treated with PBS, Mag, Mag^RGD^, _I_Mag or _I_Mag^RGD^ with NIR laser irradiation as described in Fig. 6A, and tumor tissues were harvested from different groups at the end of 16 days of treatment. Data are expressed as means ± SD (n = 5). (For interpretation of the references to colour in this figure legend, the reader is referred to the Web version of this article.)

The promising therapeutic effect of _I_Mag^RGD^ was ascribed to relieved immunosuppressive tumor microenvironment. First, the polarization of TAMs in tumor tissue was assessed via flow cytometry. As revealed in Fig. 7A and B, under NIR laser irradiation, treatments with Mag, Mag^RGD^, _I_Mag and _I_Mag^RGD^ could reduce the percentage of M2 macrophages and increase the percentage of M1 macrophages in tumor tissue compared with treatment with PBS, where _I_Mag^RGD^ had the strongest effect on the polarization of TAMs and induced the highest ratio of M1/M2 (Fig. 7C). However, without NIR laser irradiation, these treatments with various Mags only showed a slight change in the frequencies of M2 macrophages and M1 macrophages of the tumor tissues compared with treatment with PBS (Figs. S21A and B). Furthermore, the cytokines (IL-10, TNF-α and IFN-γ) of tumor tissue lysates and serum were examined after different treatments. The results showed that treatment with _I_Mag^RGD^ could most significantly reduce the secretion of IL-10 (Fig. 7D), and elevate the secretion of TNF-α and IFN-γ in tumor tissue lysates with NIR laser irradiation (Fig. 7E and F). The secretion of IL-10, TNF-α and IFN-γ in the serum exhibited a similar trend (Figs. S22A–C). However, without NIR laser irradiation, all these treated mice remained the high level of IL-10, the low level of TNF-α and IFN-γ in the tumor tissue lysates and serum (Figs. S23A–F). In terms of the important role of IFN-γ [45], the immunofluorescence staining on tumor tissue section was further performed to investigate the intratumoral level of IFN-γ, confirming the highest intratumoral level of IFN-γ (labeled by TRITC) in _I_Mag^RGD^ group with NIR laser irradiation (Fig. S24) and the low level of IFN-γ in all the groups without NIR laser irradiation (Fig. S25). These results confirm that the _I_Mag^RGD^ can induce TAMs repolarization with NIR laser irradiation, which promotes the tumor immunotherapeutic efficacy. Since TNF-α and IFN-γ promote DC maturation [46], the frequency of mature DCs (CD80^+^ CD86^+^) in lymph node was investigated by flow cytometry. As shown in Figs. S26A and B, the percentage of mature DCs increased slightly within lymph node after treatment with _I_Mag^RGD^ without NIR laser irradiation. Meanwhile, with NIR laser irradiation, treatment with _I_Mag^RGD^ exhibited more significant increase in the frequency of mature DCs in lymph node (Fig. 7G and S27). In addition, T cell activation-dependent antitumor immune response was also evaluated by immunofluorescence. The results showed a significant boost of tumor-infiltrating T helper cells (CD4^+^ labeled by TRITC) and cytotoxic T lymphocytes (CD8^+^ labeled by FITC) in tumor of mice treated with _I_Mag^RGD^ under NIR laser irradiation (Fig. 7H). Moreover, the corresponding flow cytometry analysis also proved that the percentages of tumor-infiltrating T helper cells (CD4^+^) and cytotoxic T lymphocytes (CD8^+^) in tumor of _I_Mag^RGD^ group were highest among all the groups (Fig. S28). However, without NIR laser irradiation, the immunofluorescence images and flow cytometry analysis revealed that all the treated mice remained the low percentages of CD4^+^T cells and CD8^+^ T cells (Figure S29 and S30A-C). Therefore, _I_Mag^RGD^ under NIR laser irradiation can repolarize TAMs from M2 type into M1 type, enhance DCs maturation, and increase T helper cells and cytotoxic T lymphocytes infiltration into tumor site, relieving the immunosuppressive tumor microenvironment and leading to the promising therapeutic effect.

## 3 Conclusion

In this study, we incorporated IR780 and c(RGDyK) into a highly biocompatible Mag to form _I_Mag^RGD^ with the capability of PDT/PTT and TAMs repolarization in tumor situ. The vitro and in vivo results demonstrated that _I_Mag^RGD^-induced PDT/PTT could damage tumor cells and cause ICD via hyperpyrexia and ROS. Furthermore, _I_Mag^RGD^ promoted the repolarization of TAMs from M2 type to M1 type, increased the percentage of DC maturation and enhanced infiltration of T helper cells (CD4^+^) and cytotoxic T lymphocytes (CD8^+^) in the tumor tissues. These properties allow _I_Mag^RGD^ to transform the tumor microenvironment from immunosuppressive to immunostimulative, thus effectively controlling tumor growth. In addition, _I_Mag^RGD^ could be used for fluorescence imaging and magnetic imaging of tumors. Therefore, this work provides an efficient and safe strategy for photoimmunotherapy and diagnosis of tumors.

## 4 Materials and methods

1. Materials

1,2-distearoyl-sn-glycero-3-phosphoethanolamine-N-[methoxy(polyethylene glycol)-2000]-c(RGDyK) (DSPE-PEG2000-cRGDyK) was obtained from Ruixi, Xi'an (China). IR780, 3-(4,5-Dimethylthiazol-2-yl)-2,5-diphenyltetrazolium bromide (MTT) and 9,10-phenanthrenequinone were obtained from Aladdin Co. Ltd. (Shanghai, China). PI and Annexin-FITC assay kit were acquired from Yuanye Bio-Technology (Shanghai, China).

2. Preparation of Mag

*Magnetospirillum gryphiswaldense MSR-1* (DSM 6361) was purchased from Deutsche Sammlung von Mikroorganismenund Zellkulturen GmbH (Brunswick, Germany). 5 mL of MSR-1 were cultured in 50 mL of schleifer liquid medium with 0.06 mM ferric citrate in a stoppered 100 mL-bottle containing 5 % O_2_ and 95 % N_2_ in the gas phase and grown orbital shaker at a speed of 100 r/min for 4 d at 30 °C [34]. Then *MSR-1* were harvested through centrifugation at 8000 rpm for 15 min and suspended in PBS (0.01 mol L^−1^, pH 7.4) for 25 min of sonication with a time interval of 5 s at 200 W. Afterwards, the Mag was magnetically separated using a magnet and washed with PBS three times.

3. Preparation of _I_Mag^RGD^

100 μL of 20 mg mL^−1^ Mag suspension in PBS solution was mixed with 100 μL of 3 mg mL^−1^ IR780 in DMSO, 100 μL of 2 mg mL^−1^ DSPE-PEG2000-cRGDyK in DMSO and 700 μL of 4-(2-hydroxyethyl)-1-piperazineethanesulfonic acid buffer. The resulting mixture was sonicated in an ice-water bath for 30 min with 1 min pulse for every 1 min and incubated overnight in an orbital shaker (60 rpm) [47]. _I_Mag^RGD^ was then collected by using a magnet and washed with PBS (0.01 mol L^−1^, pH 7.4) three times. The same procedure was used to prepare _I_Mag or Mag^RGD^ in the presence of IR780 or DSPE-PEG2000-cRGDyK alone.

4. Characterization

Surfer charge and hydrodynamic sizes of different Mags were determined by Malvern Zetasizer (Nano ZS, Malvern, USA). TEM image was captured by transmission electron microscope (JEM-2010) with 200 kV of accelerating voltage. UV–Vis absorption spectra was measured by a Shimadzu UV-3600 spectrophotometer. Fluorescence spectra was measured by a SpectraMax M5 microplate reader. XRD was measured by a Rigaku-Dmax 2500 diffractometer using Cu Ka radiation (40.0 kV, 200.0 mA).

The cRGD content of _I_Mag^RGD^ was detected by measuring arginine content using a previously described fluorimetric assay [35]. Briefly, 50 μL of 1 mg mL^−1^_I_Mag^RGD^ PBS suspension was mixed with 150 μL of 150 μM 9,10-phenanthrenequinone reagent in ethanol and 25 μL of 2 M sodium hydroxide (NaOH) aqueous solution for 3 h at 60 °C. Then, 50 μL of the reaction liquid was transferred to a 96-well plate and mixed with 50 μL (1.2 M) hydrogen chloride (HCl) aqueous solution. Fluorescence emission intensity at 395 nm was recorded by a SpectraMax M5 microplate reader at excitation of 312 nm.

IR780 content of _I_Mag^RGD^ was determined using a SpectraMax M5 microplate reader (740 nm excitation, continuous wavelength from 750 to 900 emission).

5. Photothermal and Photodynamic performance from _I_Mag^RGD^

1 mL of 100 μg mL^−1^ Mag, _I_Mag, Mag^RGD^ or _I_Mag^RGD^ PBS suspension (equivalent to Fe_3_O_4_ content) were irradiated under an 808 nm laser at the density of 0.75 W cm^−2^ for 5 min, and the temperature was measured via thermometer (DT1311, China). ^1^O_2_ level was determined by SOSG assay. Briefly, 80 μL of 12 μmol L^−1^ SOSG was mixed with 20 μL of 100 μg mL^−1^ Mag, _I_Mag, Mag^RGD^ or _I_Mag^RGD^ (equivalent to Fe_3_O_4_ content), followed by irradiation with an 808 nm laser at the density of 0.75 W cm^−2^ for 5 min or not. SOSG fluorescence emission spectra were collected at 460–640 nm with an excitation wavelength of 394 nm.

6. Cell culture

B16F10 cells and RAW264.7 cells were cultured in Dulbecco's modified Eagle medium (DMEM) supplemented with 10 % fetal calf serum, 100 units mL^−1^ penicillin and 100 units mL^−1^ streptomycin at 37 °C in humidified atmosphere with 5 % CO_2_. The medium was changed every 2 days.

7. Cell viability assessment

Cell viability was determined by MTT assay. Briefly, 100 μL of culture medium containing 1 × 10^4^ B16F10 cells were plated in each well of a 96-well plate overnight. Then, the culture medium was replaced with 100 μL of fresh medium containing various concentrations of Mag, _I_Mag, Mag^RGD^ or _I_Mag^RGD^ (equivalent to Fe_3_O_4_ content). After 6 h of incubation, the 96-well plate was irradiated with an 808 nm laser for 5 min (0.75 W cm^−2^) or not, followed by another 18 h of incubation. After treatments, the medium was replaced with 100 μL of fresh medium containing 20 μL of 5 mg mL^−1^ MTT solution. After 3.5 h of incubation in the dark, each well was added 150 μL of DMSO and the absorbance at 490 nm was measured by a Shimadzu UV-3600 spectrophotometer. For the apoptotic/necrotic cell assay, 1.6 × 10^5^ B16F10 cells were cultured in six-well plate overnight. Then, the cell medium was changed with fresh medium containing Mag, _I_Mag, Mag^RGD^ or _I_Mag^RGD^ (equivalent to 100 μg mL^−1^ Fe_3_O_4_ content) for 6 h. The plate was irradiated by an 808 nm laser for 5 min (0.75 W cm^−2^) or not, followed by another 18 h of incubation. After treatments, cells were detached by trypsin, centrifuged at 1000 rpm for 5 min and washed 3 times with PBS. The cells were suspended in PBS, stained with 5 μL of 100 μg mL^−1^ PI and Annexin-FITC, and analyzed by flow cytometry (BD Accuri™ C6 plus).

8. Cellular ROS level detection

1 × 10^5^ B16F10 cells were seeded into each well of a six-well plate overnight. Cells were treated with Mag, _I_Mag, Mag^RGD^ or _I_Mag^RGD^ (equivalent to 100 μg mL^−1^ Fe_3_O_4_ content) for 6 h, followed by 5 min of 808 nm laser irradiation (0.75 W cm^−2^) or not. After another 6 h of incubation, the cells were washed three times by PBS, and incubated with 10 μmol L^−1^ of H2DCFDA at 37 °C for 30 min. Then, the fluorescence image was acquired with Nikon fluorescence microscope (Tokyo, Japan) and fluorescence intensity was analyzed by flow cytometry (BD Accuri™ C6 plus).

9. Cellular uptake

1.5 × 10^5^ B16F10 cells were seeded in each well of a six-well plate overnight, and then incubated with free IR780, _I_Mag or _I_Mag^RGD^ (equivalent to 10 μg mL^−1^ IR780 content) for 3 h. Then, the cells were washed with PBS three times. Cell uptake was analyzed by fluorescence microscopy (Zeiss Axio Vert A1, Germany).

10. Assessment of DCs maturation in DCs/B16F10 transwell system

1 × 10^5^ B16F10 cells were seeded into the lower chamber of six-well transwell plate (Corning; polyester membrane; 0.4 μm pore size; 6.5 mm diameter; 0.33 cm^2^ membrane surface area). After 24 h of incubation, B16F10 cells in the lower chamber were treated with Mag, _I_Mag, Mag^RGD^ or _I_Mag^RGD^ (equivalent to 100 μg mL^−1^ Fe_3_O_4_ content), and 1 × 10^4^ DCs were seeded into the upper chamber. After 6 h of treatment, the lower chamber was irradiated with an 808 nm laser (0.75 W cm^−2^, 5 min) or not, followed by additional 18 h of incubation. The cells in the upper chamber were collected, washed three time with PBS, and stained with anti-CD80-FITC (1 μg mL^−1^, clone 16-10A1, Biolegend) and anti-CD86-PE (1 μg mL^−1^, clone GL-1, Biolegend) for 30 min, respectively. The DCs mature level was analyzed by flow cytometer (BD Accuri™ C6 plus). And the supernatant was centrifuged at 3000 rpm for 10 min to remove cell debris or dead cell, and the levels of IL-10, TNF-α, IL-6 and IL-12 were assessed by ELISA kit (Biolegend) according to manufacturer's instruction.

11. Macrophage repolarization assessment

1 × 10^6^ RAW264.7 cells were incubated with IL-4 (100 ng mL^−1^) for 24 h to generate M2 macrophages. 1 × 10^5^ B16F10 cells were seeded into the lower chamber of six-well transwell plate (Corning; polyester membrane; 4 μm pore size; 6.5 mm diameter; 0.33 cm^2^ membrane surface area). Then, 1 × 10^5^ M2 macrophages were seeded into the upper chamber. And, Mag, Mag^RGD^, _I_Mag or _I_Mag^RGD^ (equivalent to 100 μg mL^−1^ Fe_3_O_4_ content) was added to both chambers for 6 h, followed by an 808 nm laser irradiation (0.75 W cm^−2^) for 5 min to the lower chamber or not. After another 18 h incubation, the treated M2 macrophages were collected and labeled with anti-F4/80-FITC (0.5 μg mL^−1^, clone BM8, Biolegend), anti-CD206-APC (1 μg mL^−1^, clone C068C2, Biolegend), and anti-CD86-PE (1 μg mL^−1^, clone GL-1, Biolegend). Flow cytometer (BD Biosciences, AccuriC6) was applied to analyze the percentages of M1 and M2. And the supernatant was centrifuged at 3000 rpm for 10 min to remove cell debris or dead cell, and the levels of IL-10, TNF-α and IFN-γ were assessed by ELISA kit (Biolegend) according to manufacturer's instruction.

12. Establishment of the B16F10 tumor-bearing mice model

Female C57BL/6J mice (4–6-weeks-old) were purchased from the Animal Experimental Center of Jilin University (Changchun, China) and kept under thermo-regulated, humidity-controlled conditions under a 12 h day/night light cycle provided by the experimental. Mice were fed with standard rat chow and water ad libitum. The back of each mouse was subcutaneously inoculated 1 × 10^6^ B16F10 cells in 100 μL of PBS. All animal studies were carried out in Changchun Institute of Applied Chemistry, Chinese Academy of Sciences, and the operating procedures of the experimental animals were carried out in accordance with protocols approved by the Committee for Animal Research of Changchun Institute of Applied Chemistry, Chinese Academy of Sciences China.

13. In vivo biodistribution

When the tumor gets to a volume of ∼100 mm^3^, B16F10 tumor-bearing mice were intravenously injected with _I_Mag, _I_Mag^RGD^ or free IR780 (equivalent to 0.25 mg kg^−1^ IR780 content). After 24 h, the whole-body fluorescence images of mice were captured using in vivo fluorescence imaging system (IVIS® Spectrum system, Caliper, Hopkinton, MA, USA). After the fluorescence imaging, the mice were sacrificed, and the major organs (heart, liver, spleen, kidney, lung) and tumors were excised for quantification of fluorescence intensity. Furthermore, the main organ and tumors were weighed and predigested with aqua regia for ICP-OES analysis (ThermoScientific iCAP6300). The data were expressed as a percentage of the injected dose per gram of tissue (%ID/g). To acquire the T1-weighted magnetic resonance imaging, we used a Bruker 7.0 T MR imaging system and a spin-echo method and adopted the following parameters: time to echo (TE), 11.5 ms; repetition time (RE), 400 ms; number of excitations (NEX), four; slice thickness, 1 mm; field of view (FOV), 44 × 44 mm^2^; matrix size, 256 × 256.

14. In vivo therapeutic evaluation

When the tumor gets to a volume of ∼100 mm^3^, B16F10 tumor-bearing mice were divided into 10 groups. 5 groups were intravenously injected with PBS, Mag, _I_Mag, Mag^RGD^ and _I_Mag^RGD^ (equivalent to 10 mg kg^−1^ Fe_3_O_4_), respectively. At 12 h post-injection, the tumor of mice was irradiated with an 808 nm laser at the power intensity of 0.75 W cm^−2^ for 5 min. Another 5 groups were similarly treated but without NIR laser irradiation. The mice were treated every day for total three days. The temperature of tumor area was monitored by infrared camera (FLIR, USA). The tumor volume of mice was measured by a caliper and calculated by a formula of volume = ab^2^/2 (a and b are the tumor length and width respectively). The tumors were sectioned and stained by H&E. The images were captured by a digital microscope (Nikon Eclipse 80i). In order to analyze the apoptosis/necrosis of tumor cells, tumor tissues were obtained from each group on the 16th day. Tumor tissues were cleaned with cold PBS and incubated in DMEM containing Collagenase A (2 mg mL^−1^) and DNase I (50 units mL^−1^). The single cells were acquired by using 70 μm nylon strainers to filter. The single cells were suspended in PBS, stained with 5 μL of 100 μg mL^−1^ PI and Annexin-FITC, and analyzed by flow cytometry (BD Accuri™ C6 plus).

15. Intratumoral macrophage, intralymph node DCs and intratumoral T cell analysis

For intratumoral macrophage analysis, the single cells of tumors were stained with anti-F4/80-FITC (1 μg mL^−1^, clone BM8, Biolegend), anti-CD206-APC (1 μg mL^−1^, clone C068C2, Biolegend), and anti-CD86-PE (1 μg mL^−1^, clone GL-1, Biolegend) for 30 min. Then, the samples were detected by flow cytometry. For DCs analysis, the single cells of lymph node were stained with anti-CD80-FITC (1 μg mL-1, clone 16-10A1, Biolegend) and anti-CD86-PE (1 μg mL-1, clone GL-1, Biolegend) for 30 min. Then, the samples were detected by flow cytometry. For intratumoral T cell flow cytometry analysis, the single cell suspension of tumors was stained with anti-CD3-FITC (1 μg mL-1, clone 17A2, Biolegend), anti-CD4-PE (1 μg mL-1, clone GK1.5, Biolegend) and anti-CD8-APC (1 μg mL-1, clone 53-6.7, Biolegend) for 30 min. Then, the samples were detected by flow cytometry. For intratumoral T cells immunofluorescence analysis, the tumors were extracted. OCT-embedded frozen tumors were sectioned (5 μm), fixed in 4 % paraformaldehyde for 10 min, and permeabilized with Triton 0.01 % for 15 min, and then blocked with PBS containing 5 % FBS for 1 h. Sections were incubated with CD4 antibody (1:200, Abcam) and CD8 antibody (1:200, Abcam) overnight at 4 °C, and then with species-specific secondary antibodies coupled with FTIC and TRITC (Invitrogen) for 1 h at room temperature. The fluorescent signals of section were detected under fluorescence microscopy (Nikon Eclipse 80i).

16. The intratumoral colocalization analysis of _I_Mag^RGD^ with macrophages and tumor cells

The tumor tissues were collected at 48 h post-injection of IR780, _I_Mag or _I_Mag^RGD^ for preparation of tumor sections. Then, sections were incubated with F4/80 antibody (1:200, Biolegend) or HMB45 antibody (1:200, Abclonal) overnight at 4 °C, and then with species-specific secondary antibodies coupled with FTIC (Invitrogen) for 1 h at room temperature. The fluorescent signals of section were detected under fluorescence microscopy (Nikon Eclipse 80i).

17. The intratumoral level of IFN-γ analysis

The tumors were extracted at the end of 16 days of treatment for preparation of tumor sections. Sections were incubated with IFN-γ antibody (1:200, Nature Biosciences) overnight at 4 °C, and then with species-specific secondary antibodies coupled with TRTIC (Invitrogen) for 1 h at room temperature. The fluorescent signals of section were detected under fluorescence microscopy (Nikon Eclipse 80i).

18. Toxicity evaluation in vivo

The body weight of each mouse was measured every other day for total 16 days. Peripheral blood samples of mice in different groups were taken from the eye socket at the end of treatment and centrifuged at 800 g for 10 min. The plasma fraction was re-centrifuged at 800 g for 10 min again to obtain cell free serum. The aspartate transaminase (AST), alanine transaminase (ALT), creatinine (CREA) and blood urea nitrogen (BUN) levels of the serum were detected using standard kits (Nanjing Institute of Biological Engineering, China) according to the manufacturer's protocol. In addition, the mice were sacrificed and the major organs (including heart, liver, spleen, lung and kidney) were collected and sectioned for H&E staining. The images were captured by a digital microscope (Nikon Eclipse 80i).

19. Statistical analysis

All data analyses were performed using GraphPad Prism 7.0 (GraphPad Software, La Jolla, CA, USA). Data are presented as the mean ± SD. Student's t-test was used to analyze differences between two groups. One-way ANOVA was used to perform the multi-sample analysis followed by the Tukey post hoc test. Differences at p < 0.05 were considered statistically significant (∗p < 0.05; ∗∗p < 0.01; and ∗∗∗p < 0.001; ns, not significant).

## CRediT authorship contribution statement

**Xiaoqing Han:** Writing – original draft, Methodology, Investigation, Formal analysis, Conceptualization. **Xingbo Wang:** Supervision, Investigation. **Jiao Yan:** Methodology, Investigation. **Panpan Song:** Methodology, Investigation. **Yanjing Wang:** Methodology, Investigation. **Yaqing Kang:** Methodology, Investigation. **Abdur Rauf:** Supervision. **Haiyuan Zhang:** Writing – review & editing, Visualization, Supervision, Funding acquisition, Conceptualization.

## Ethics approval and consent to participate

The animal experimental operation was performed in Changchun Institute of Applied Chemistry, Chinese Academy of Sciences, and the operating procedures of the experimental animals were carried out in accordance with protocols approved by the Committee for Animal Research of Changchun Institute of Applied Chemistry, Chinese Academy of Sciences China (Approval No.202071)

## Declaration of competing interest

The authors declare that they have no known competing financial interests or personal relationships that could have appeared to influence the work reported in this paper.

## Data Availability

Data will be made available on request.
