# Supplementary material for: Multifunctional biosynthesized magnetosome for multimodal imaging and combined therapy of tumor
Source: Mater Today Bio. 2024 Dec 24;30:101429. doi: 10.1016/j.mtbio.2024.101429 (PMC11750283; doi:10.1016/j.mtbio.2024.101429)
Supplement: Multimedia component 1 [file mmc1.pdf]

Supporting Information

Multifunctional biosynthesized magnetosome for multimodal imaging and combined therapy of tumor

Xiaoqing Han[a,b], Xingbo Wang[c], Jiao Yan[c], Panpan Song[c], Yanjing Wang[c], Yaqing Kang[c], Abdur Rauf[d], Haiyuan Zhang[b,c,*]

[a] Key Laboratory of Molecular Epigenetics of the Ministry of Education (MOE), Northeast Normal University, Changchun, China

[b] Laboratory of Chemical Biology, Changchun Institute of Applied Chemistry, Chinese Academy of Sciences, Changchun 130022, China

[c] School of Biomedical Engineering & The First Affiliated Hospital, Guangzhou Medical University, Guangzhou 511436 China

[d] Department of Chemistry, University of Swabi, Ambar 23430 Pakistan

*Corresponding author:

Haiyuan Zhang, Laboratory of Chemical Biology, Changchun Institute of Applied Chemistry, Chinese Academy of Sciences, Changchun 130022, China; School of Biomedical Engineering & The First Affiliated Hospital, Guangzhou Medical University, Guangzhou, China; E-mail: zhangh@ciac.ac.cn

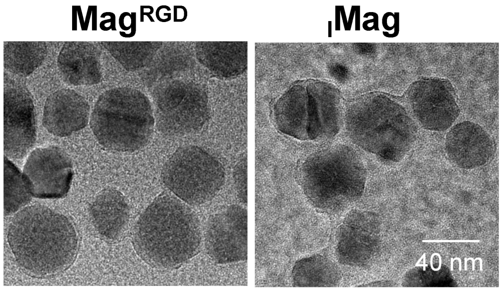

Figure S1. TEM images of Mag^{RGD} and $_{I}$Mag.

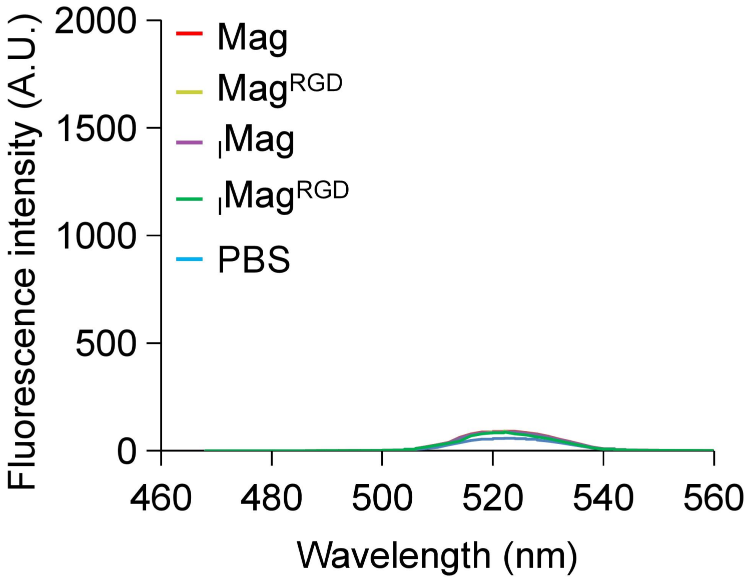

Figure S2. SOSG fluorescence emission spectra of Mag, Mag^{RGD}, $_{I}$Mag and $_{I}$Mag^{RGD} (100 μg mL^{-1} equivalent to Fe_3O_4) without NIR laser irradiation.

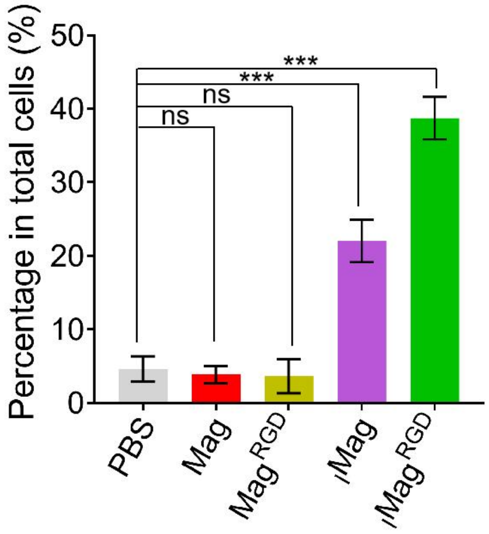

Figure S3. Quantification of intracellular ROS level in B16/F10 cells as shown in

Figure 3B. Data are expressed as means ± SD (n=3).

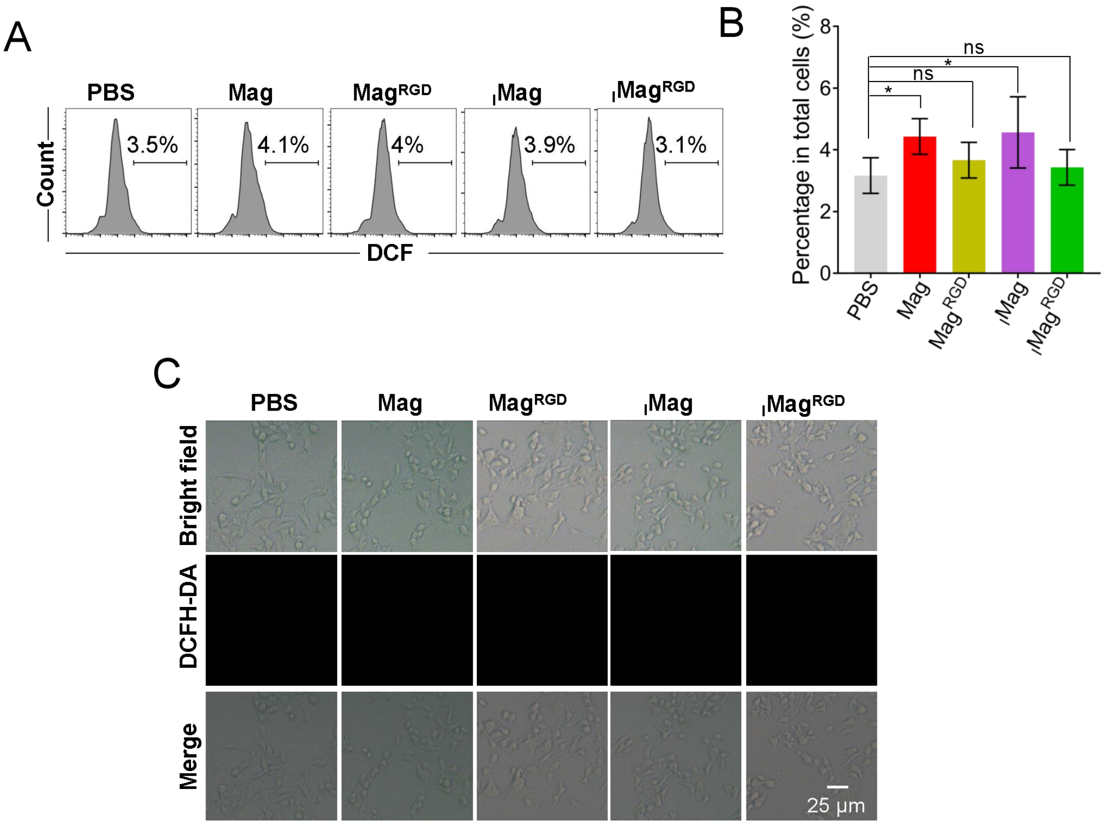

Figure S4. DCF-based intracellular ROS evaluation in B16F10 cells treated with different Mag groups (100 μg mL^{-1} equivalent to Fe_3O_4 content) without NIR laser irradiation. (A) Flow cytometry analysis for the intracellular ROS level of B16/F10 cells. (B) Quantification of intracellular ROS level in B16/F10 cells in Figure S4A. (C) Fluorescence microscopy images to show the intracellular ROS level of B16/F10 cells. Data are expressed as means ± SD (n=3).

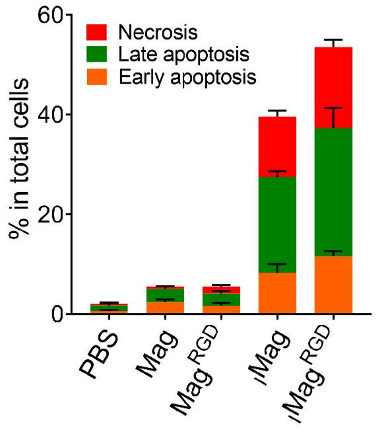

Figure S5. Quantification of early apoptosis, late apoptosis and necrosis of B16/F10 cells in Figure 3D. Data are expressed as means ± SD (n=3).

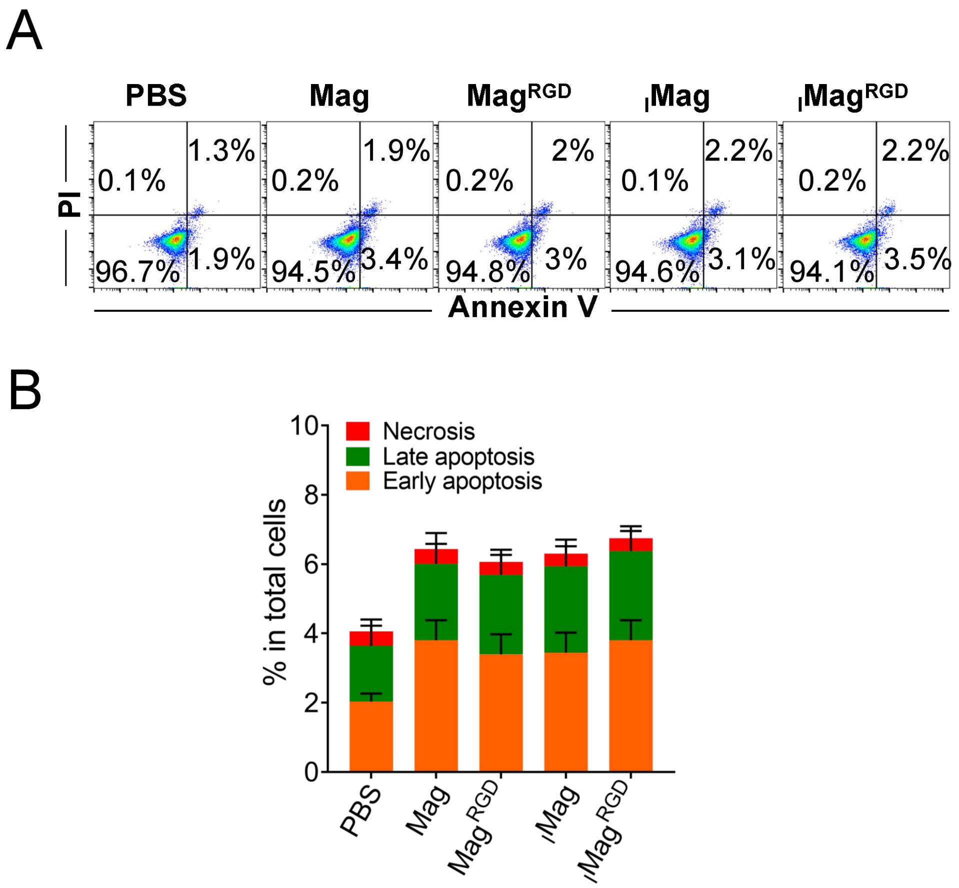

Figure S6. (A) Flow cytometry apoptosis analysis for B16/F10 cells after treatments with different Mags (100 μg mL^{-1} equivalent to Fe_3O_4 content) without laser irradiation. The B16/F10 cells were stained with Annexin-FITC and PI. (B)

Quantification of early apoptosis, late apoptosis and necrosis of B16/F10 cells in Figure S6A. Data are expressed as means ± SD (n=3).

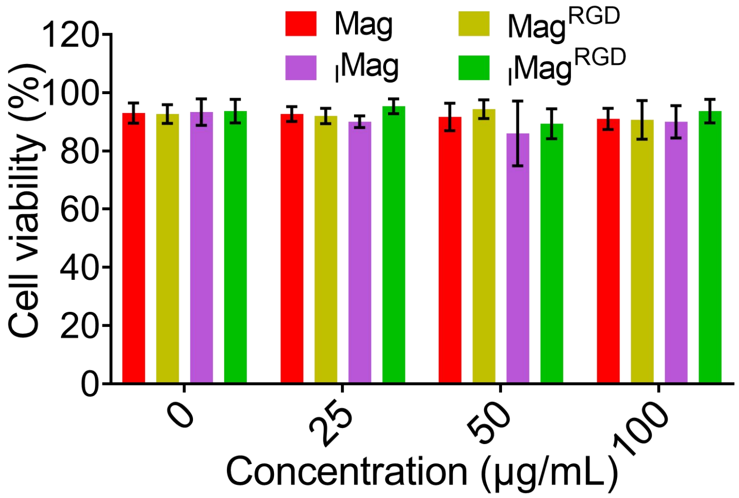

Figure S7. MTT-based viability assessment of B16/F10 cells after 24 h of treatment with different concentrations of different Mags (equivalent to Fe_3O_4 content) without laser irradiation. Data are expressed as means ± SD (n=3).

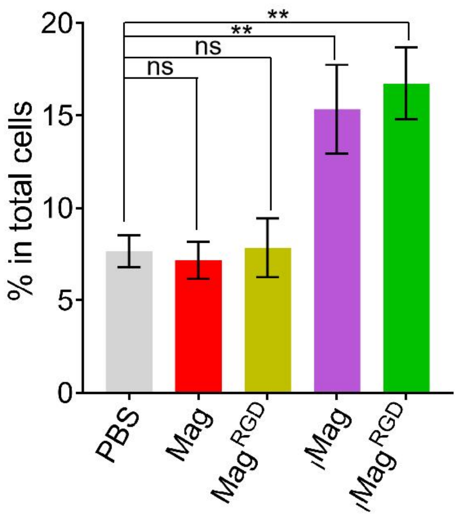

Figure S8. Quantification of mature DCs ($CD86^+CD80^+$) in Figure 3G. Data are expressed as means ± SD (n=3).

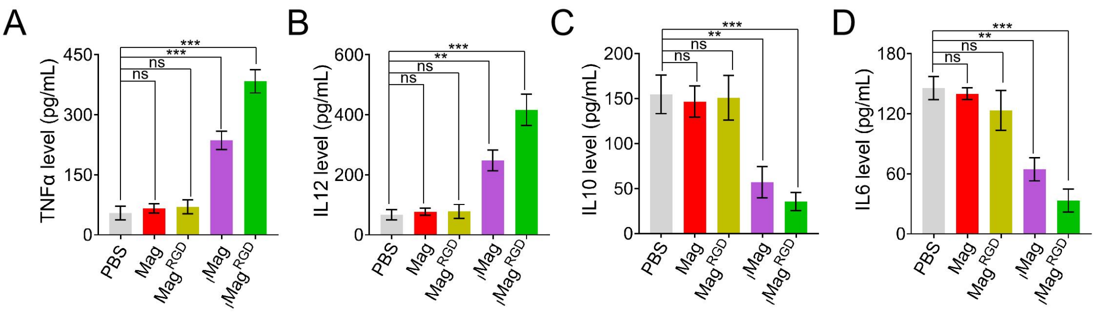

Figure S9. (A-D) ELISA analysis of the levels of TNFα (A), IL12 (B), IL10 (C) and IL6 (D) in the supernatant of the DCs/B16F10 transwell system under 808 nm laser irradiation (0.75 W cm^{-2}, 5 min) as illustrated in Figure 3F. Data are expressed as means ± SD (n=3).

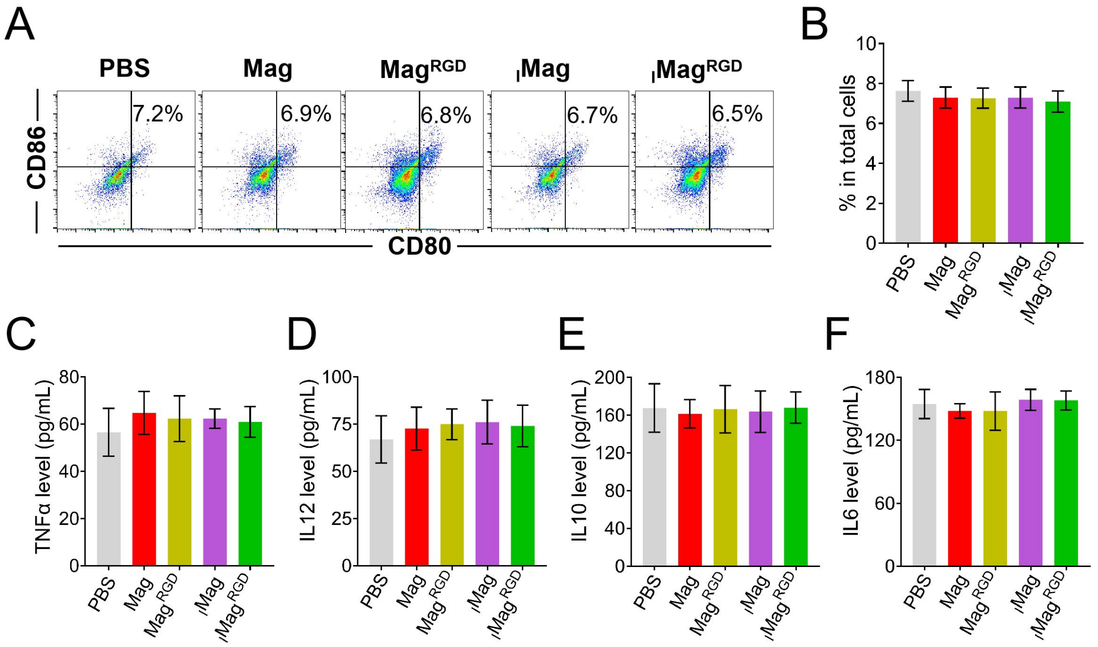

Figure S10. (A) Flow cytometry analysis for the percentage of mature DCs ($CD86^{+}CD80^{+}$). (B) Quantification of mature DCs ($CD86^{+}CD80^{+}$) in Figure 10A. (C-F) ELISA analysis of the levels of TNFα (C), IL12 (D), IL10 (E) and IL6 (F) in the supernatant of the DCs/B16F10 transwell system as illustrated in Figure 3F without NIR laser irradiation. For A to F, B16/F10 cells were treated with of different types of Mags (equivalent to 100 μg mL^{-1} Fe_3O_4 content) without NIR laser irradiation. Data

are expressed as means ± SD (n=3).

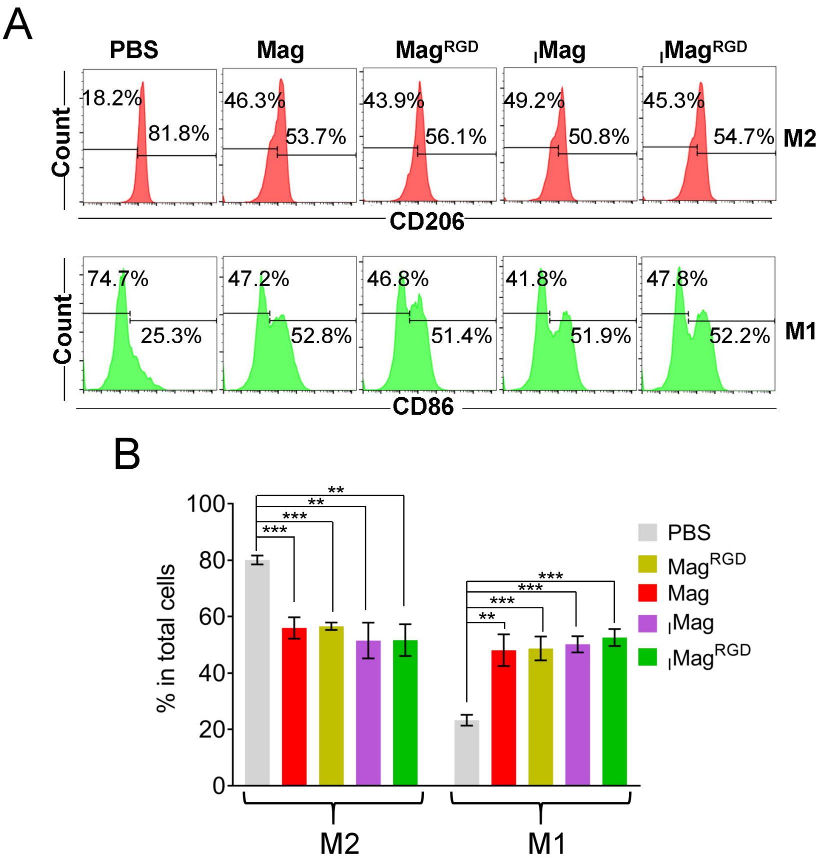

Figure S11. (A) Flow cytometric analysis of CD86 and CD206 expression in RAW264.7/B16F10 transwell system treated with different Mags (equivalent to 100 μg mL^{-1} Fe_3O_4) without laser irradiation. (B) Quantitative analysis of the percentages of M1 and M2 in Figure S10A.

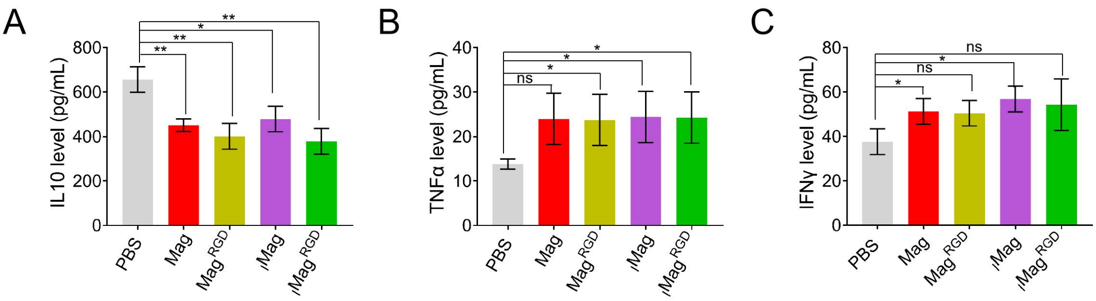

Figure S12. (A-C) ELISA analysis for the levels of IL-10 (A), TNFα (B) and IFNγ (C) secreted by RAW264.7 cells treated with different Mags (equivalent to 100 μg mL^{-1} Fe_3O_4) in RAW264.7/B16F10 transwell system without laser irradiation. Data are

expressed as means ± SD (n=3).

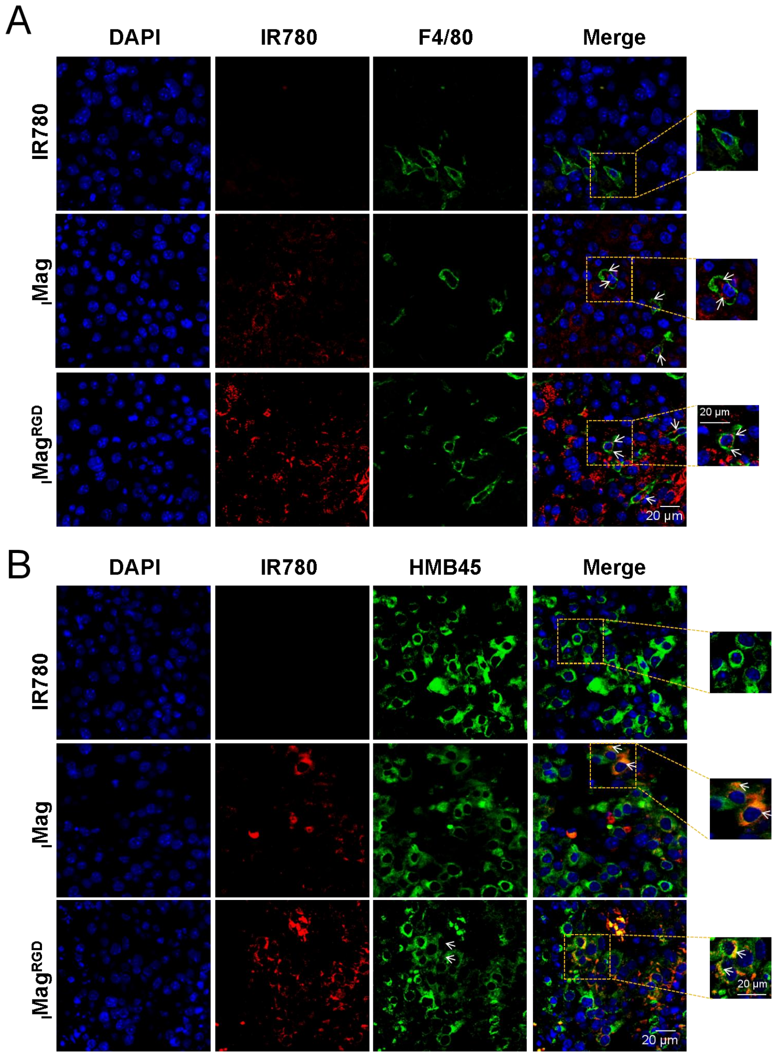

Figure S13. (A) Representative immunofluorescence images of tumor tissues stained by F4/80 (green). (B) Representative immunofluorescence images of tumor tissues stained by HMB45 (green). B16/F10 tumor-bearing mice were intravenously injected with free IR780, $_{\mathrm{I}}$Mag or $_{\mathrm{I}}$Mag^{RGD} (equivalent to 0.25 mg kg^{-1} IR780), and tumor tissues were harvested at 48 h post-injection.

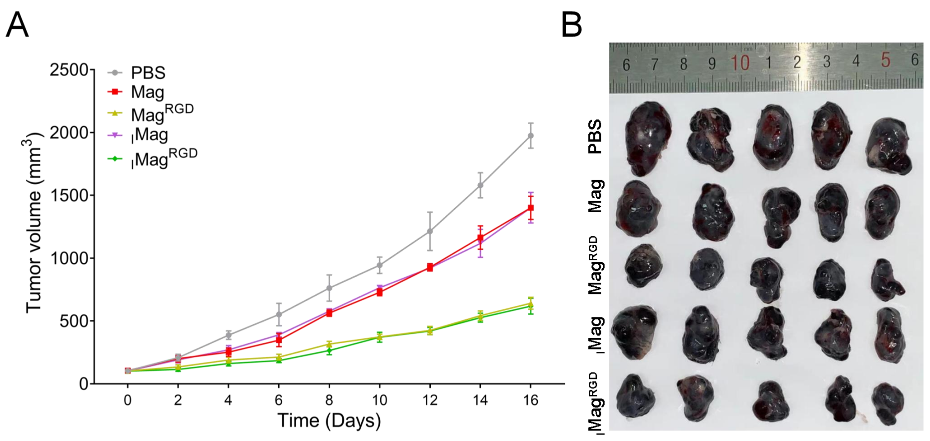

Figure S14. (A) Tumor volume curves of mice after injection with PBS, Mag, Mag^{RGD}, $_{I}$Mag or $_{I}$Mag^{RGD} without laser irradiation. (B) Photograph of tumor tissues at the end of 16 days of treatment without laser irradiation Data are expressed as means ± SD (n=5).

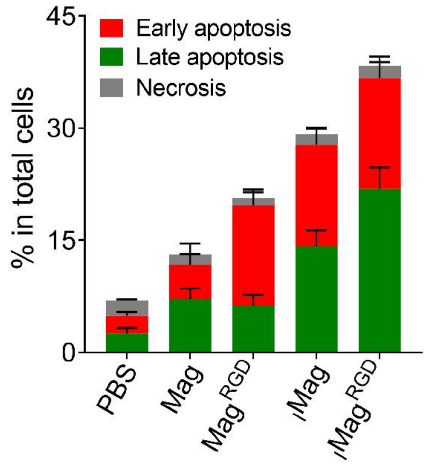

Figure S15. Quantification of early apoptosis, late apoptosis and necrosis cells in Figure 6D. Data are expressed as means ± SD (n=5).

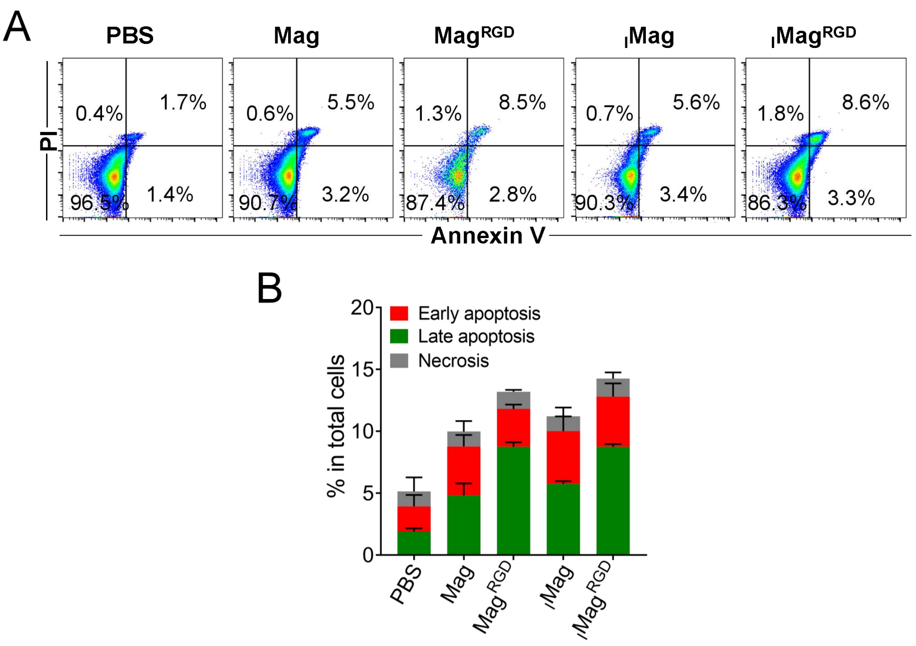

Figure S16. (A) Flow cytometry analysis for apoptotic cells in tumors at the end of treatments with different Mags without NIR laser irradiation based on Annexin-FITC/PI staining. (B) Quantification of early apoptosis, late apoptosis and necrosis cells in Figure S15A. Data are expressed as means ± SD (n=5).

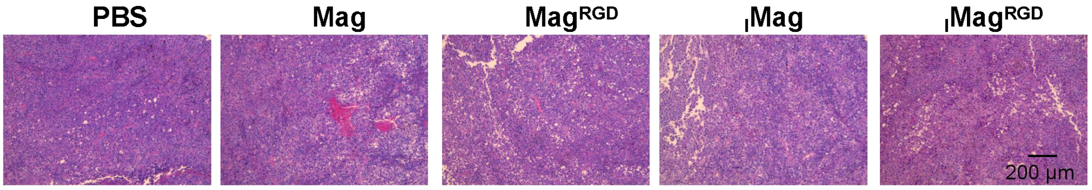

Figure S17. H&E staining of tumor tissue at the end of treatments with different Mags without NIR laser irradiation.

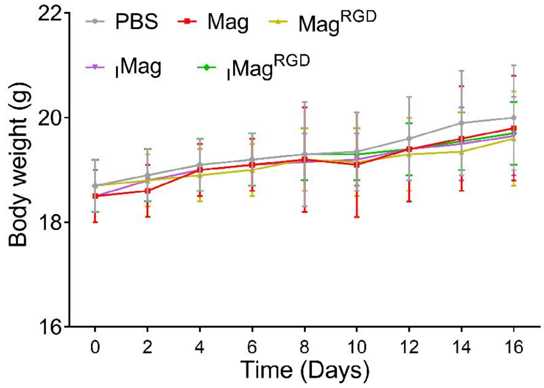

Figure S18. Body weight curves of mice in different group with NIR laser irradiation

during the treatment period. Data are expressed as means ± SD (n=5).

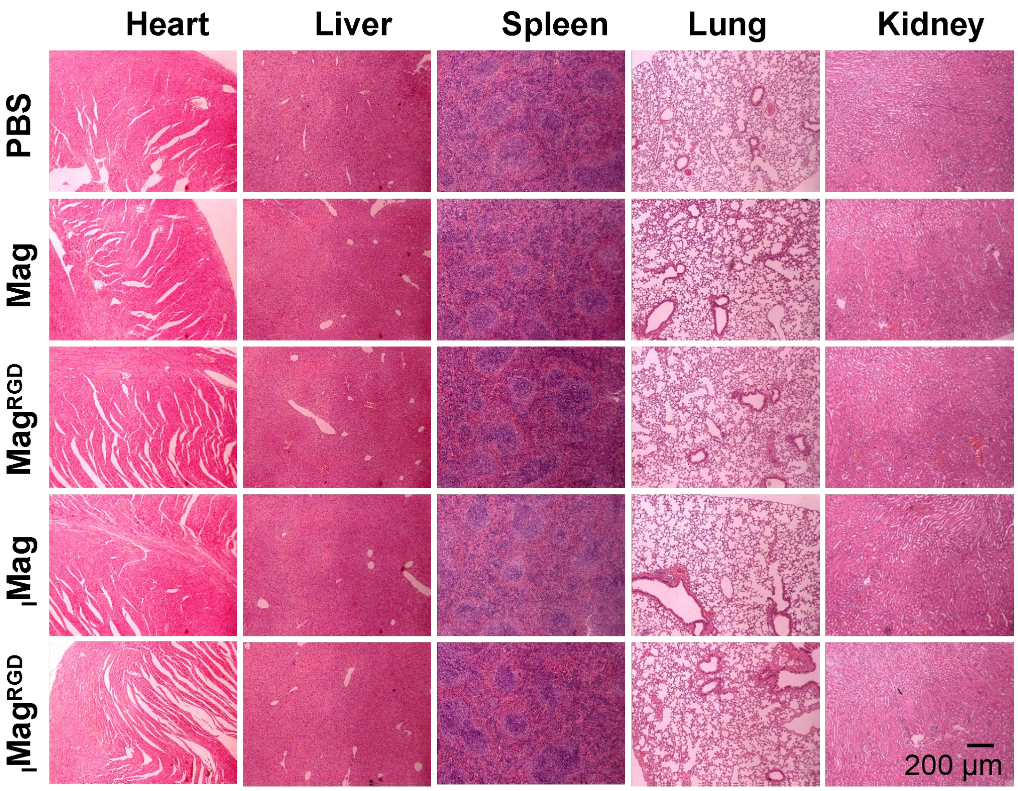

Figure S19. H&E staining of major organ tissues (heart, liver, spleen, lung and kidney) in different groups at the end of treatments with NIR laser irradiation.

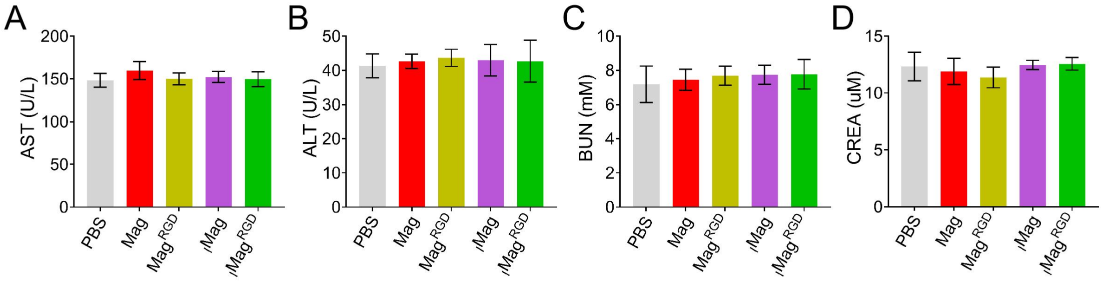

Figure S20. (A-D) Levels of AST (A), ALT (B), BUN (C) and CREA (D) in the serum of different groups at the end of treatments with NIR laser irradiation. Data are expressed as means ± SD (n=5).

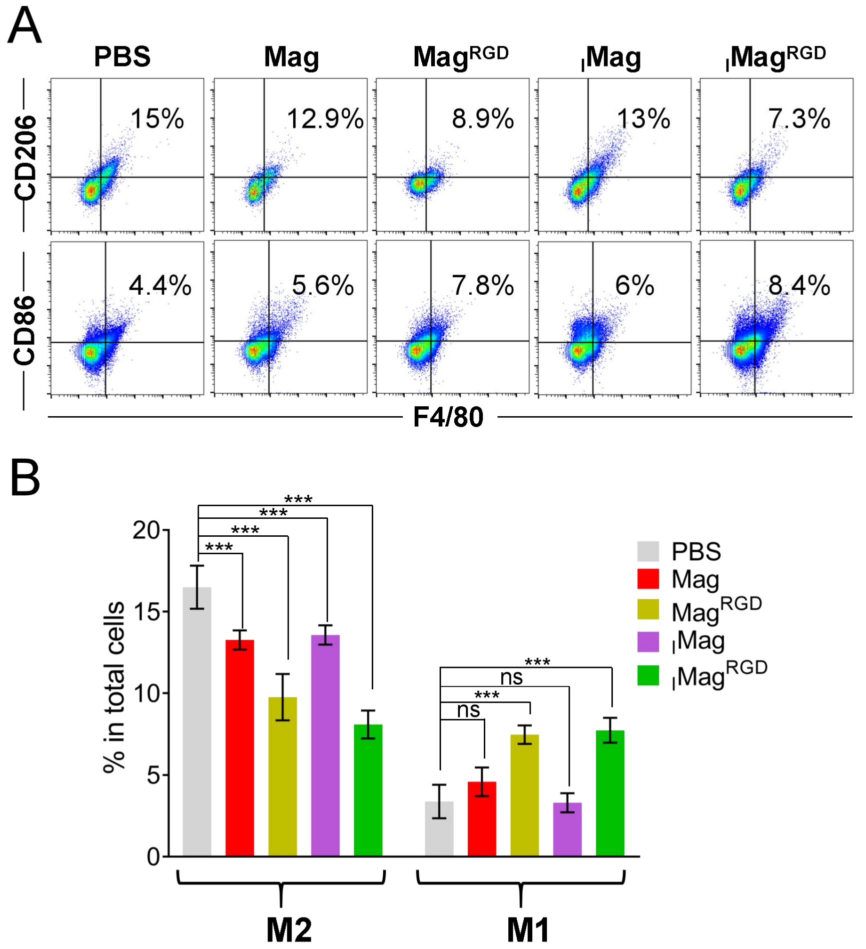

Figure S21. (A) Flow cytometric analysis of the percentages of M1-type macrophages (F4/80+CD86+) and M2-type macrophages (F4/80+CD206+) in tumor tissues of mice at the end of treatments without NIR laser irradiation. (B) Quantitative analysis of Figure S20A. Data are expressed as means ± SD (n=5).

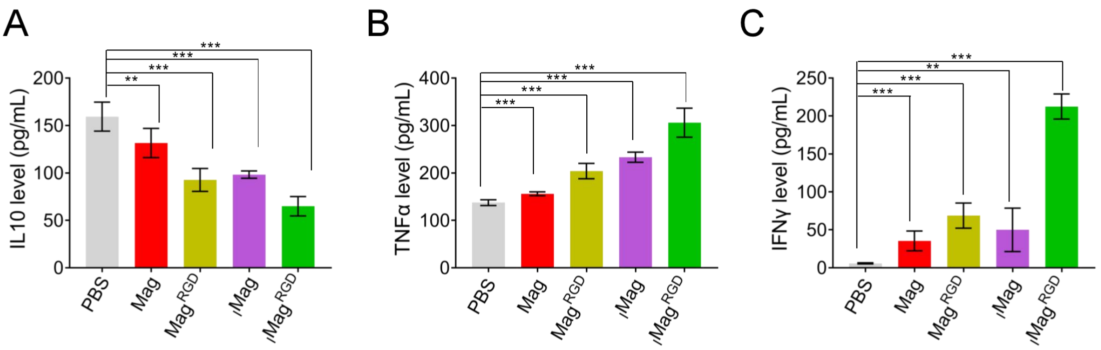

Figure S22. ELISA analysis for the levels of IL10 (A), TNFα (B) and IFNγ (C) in the serum of different groups at the end of treatments with NIR laser irradiation. Data are

expressed as means ± SD (n=5).

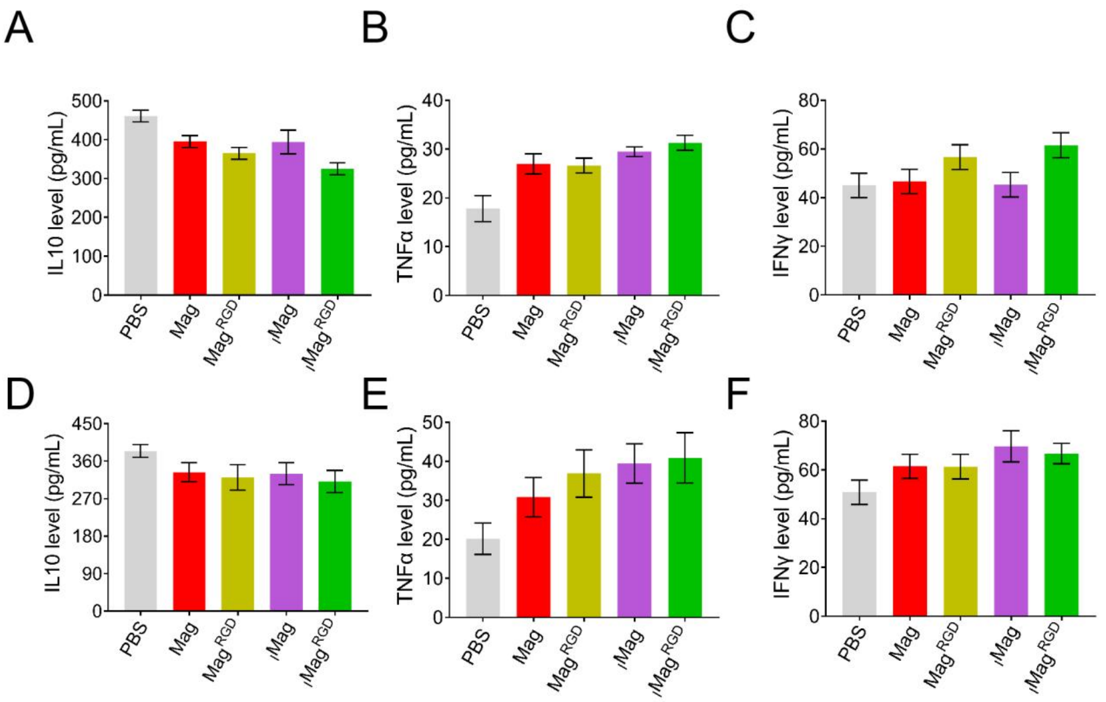

Figure S23. (A-F) ELISA analysis for the levels of cytokines in the tumor tissues and serum of different groups without NIR laser irradiation. (A: IL-10 in the tumor tissue, B: TNF-α in the tumor tissue, C: IFN-γ in the tumor tissue, D: IL-10 in the serum, E: TNF-α in the serum, F: IFN-γ in the serum). Data are expressed as means ± SD (n=5).

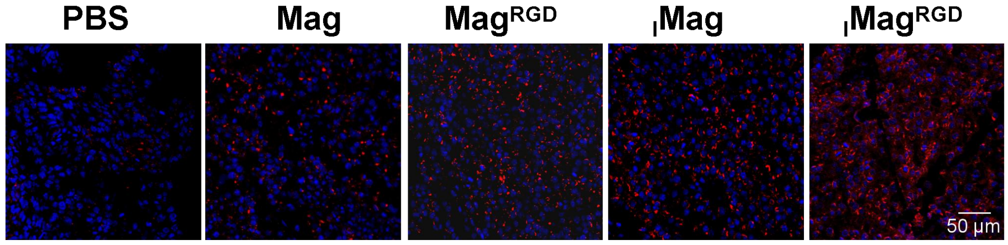

Figure S24. Representative immunofluorescence images of tumor tissues stained by IFN-γ (red). B16/F10 tumor-bearing mice were treated with PBS, Mag, Mag^{RGD}, $_{I}$Mag or $_{I}$Mag^{RGD} with NIR laser irradiation as described in Figure 6A, and tumor tissues were harvested from different groups at the end of 16 days of treatment.

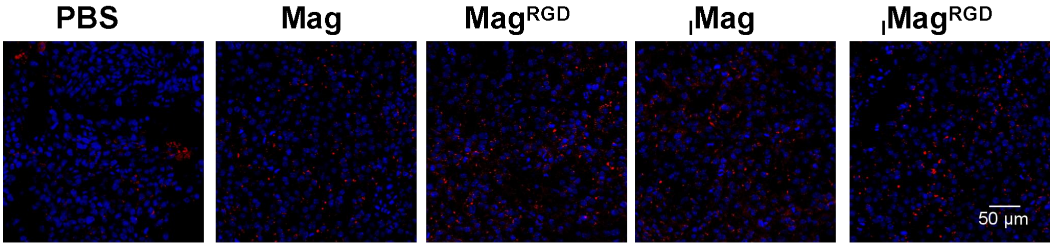

Figure S25. Representative immunofluorescence images of tumor tissues stained by IFN-γ (red) in different groups at the end of treatments without NIR laser irradiation.

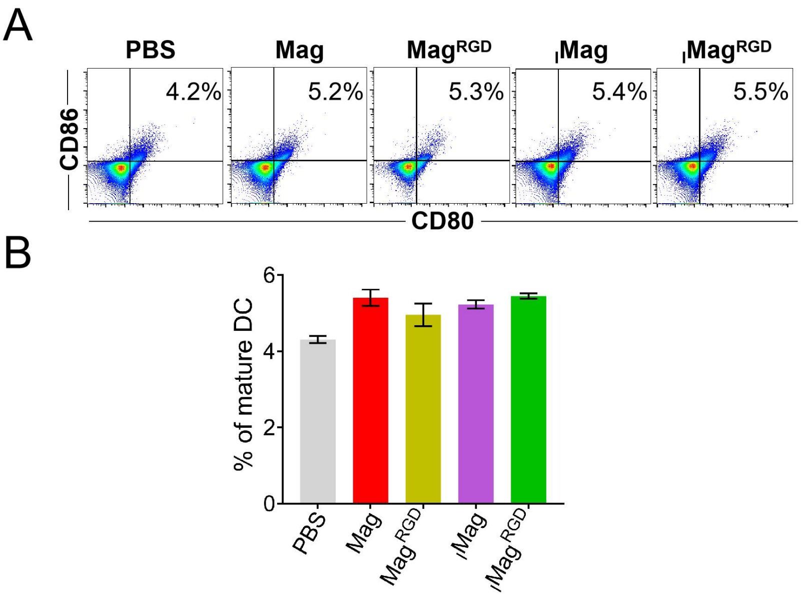

Figure S26. (A) Flow cytometry analysis for the percentage of mature DCs in draining lymph node of different groups without NIR laser irradiation. (B) Quantitative analysis of Figure S25A. Data are expressed as means ± SD (n=5).

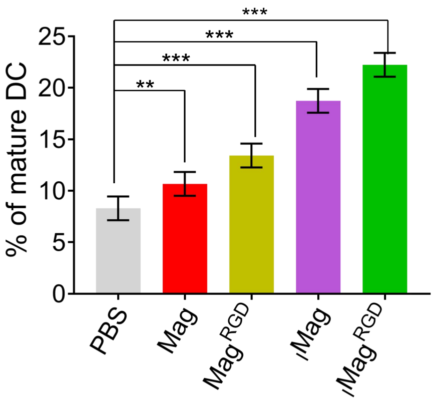

Figure S27. Quantitative analysis of Figure 7G. Data are expressed as means ± SD

(n=5).

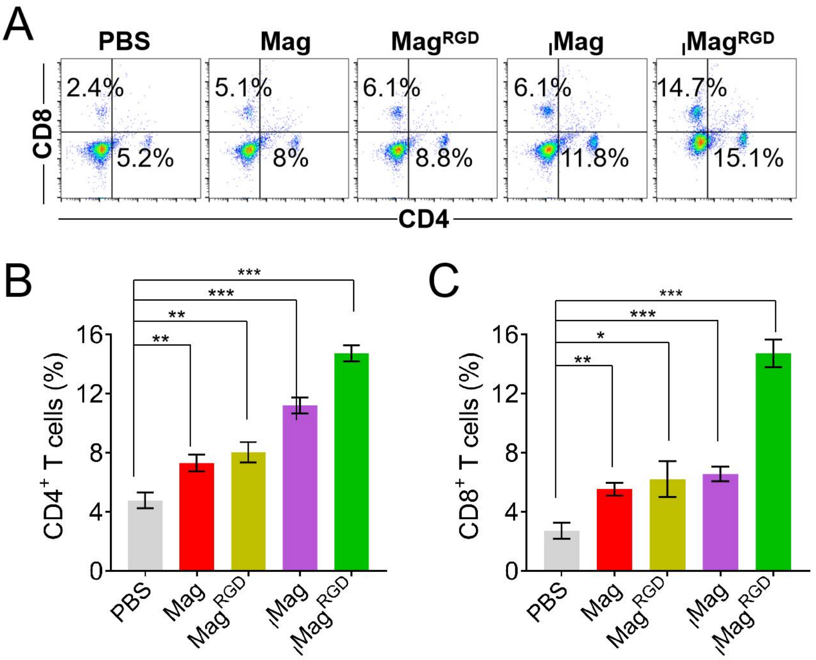

Figure S28. (A) Flow cytometry analysis for the percentages of T helper cells ($CD4^+$) and cytotoxic T lymphocytes ($CD8^+$) in tumor of different groups with NIR laser irradiation. (B and C) Quantitative analysis of Figure S27A. Data are expressed as means ± SD (n=5).

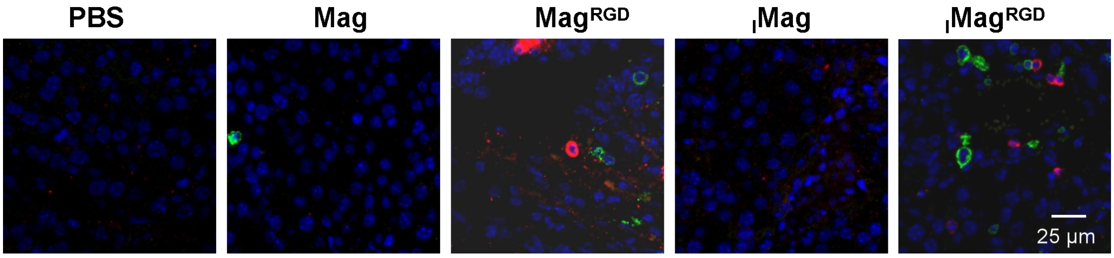

Figure S29. Representative immunofluorescence images of tumors stained by T helper cells (red, CD4) and cytotoxic T lymphocytes (green, CD8) markers at the end of treatments without NIR laser irradiation. Data are expressed as means ± SD (n=5).

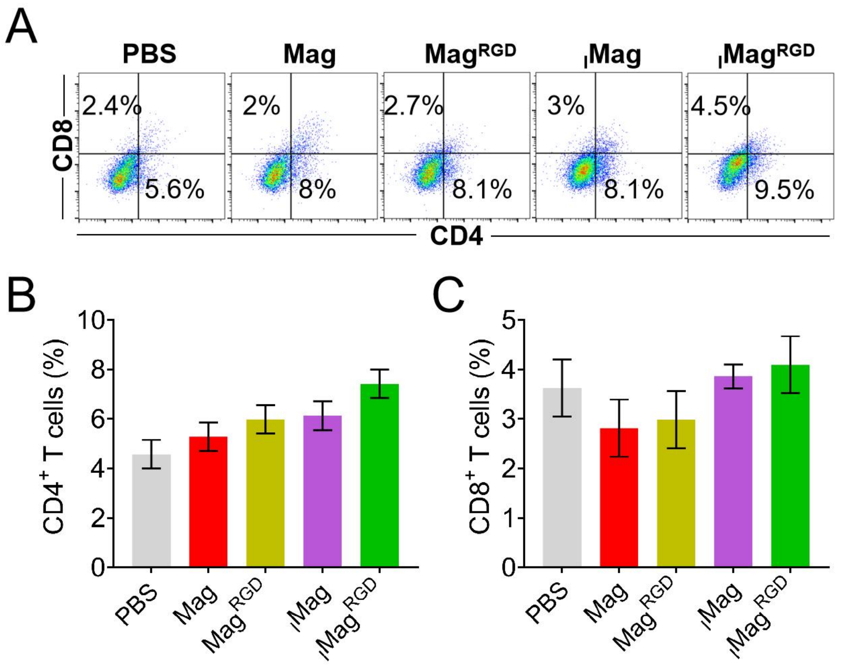

Figure S30. (A) Flow cytometry analysis for the percentages of T helper cells ($CD4^{+}$) and cytotoxic T lymphocytes ($CD8^{+}$) in tumor of different groups without NIR laser irradiation. (B and C) Quantitative analysis of Figure S29A. Data are expressed as means ± SD (n=5).
